# CRISPRi-mediated functional analysis of lung disease-associated loci at non-coding regions

**DOI:** 10.1093/nargab/lqaa036

**Published:** 2020-05-25

**Authors:** William D Stuart, Minzhe Guo, Iris M Fink-Baldauf, Alan M Coleman, John P Clancy, Marcus A Mall, Foong-Yen Lim, John J Brewington, Yutaka Maeda

**Affiliations:** 1 Division of Neonatology, Perinatal and Pulmonary Biology, Perinatal Institute, Cincinnati Children’s Hospital Medical Center, Cincinnati, OH 45229, USA; 2 Department of Pediatrics, University of Cincinnati College of Medicine, Cincinnati, OH 45229, USA; 3 Cincinnati Fetal Center, Cincinnati Children’s Hospital Medical Center, Cincinnati, OH 45229, USA; 4 Division of Pulmonary Medicine, Cincinnati Children’s Hospital Medical Center, Cincinnati, OH 45229, USA; 5 Department of Pediatric Pulmonology, Immunology and Critical Care Medicine, Charité-Universitätsmedizin Berlin, Berlin, 13353, Germany; 6 Berlin Institute of Health, Berlin, 10178, Germany; 7 German Center for Lung Research, Berlin, 13353, Germany

## Abstract

Genome-wide association studies have identified lung disease-associated loci; however, the functions of such loci are not well understood in part because the majority of such loci are located at non-coding regions. Hi-C, ChIP-seq and eQTL data predict potential roles (e.g. enhancer) of such loci; however, they do not elucidate the molecular function. To determine whether these loci function as gene-regulatory regions, CRISPR interference (CRISPRi; CRISPR/dCas9-KRAB) has been recently used. Here, we applied CRISPRi along with Hi-C, ChIP-seq and eQTL to determine the functional roles of loci established as highly associated with asthma, cystic fibrosis (CF), chronic obstructive pulmonary disease (COPD) and idiopathic pulmonary fibrosis (IPF). Notably, Hi-C, ChIP-seq and eQTL predicted that non-coding regions located at chromosome 19q13 or chromosome 17q21 harboring single-nucleotide polymorphisms (SNPs) linked to asthma/CF/COPD and chromosome 11p15 harboring an SNP linked to IPF interact with nearby genes and function as enhancers; however, CRISPRi indicated that the regions with rs1800469, rs2241712, rs12603332 and rs35705950, but not others, regulate the expression of nearby genes (single or multiple genes). These data indicate that CRISPRi is useful to precisely determine the roles of non-coding regions harboring lung disease-associated loci as to whether they function as gene-regulatory regions at a genomic level.

## INTRODUCTION

Chronic lung diseases, including asthma, cystic fibrosis (CF), chronic obstructive pulmonary disease (COPD) and idiopathic pulmonary fibrosis (IPF), are influenced by multiple genetic and environmental factors (1). In order to identify genetic loci that are linked to the pathophysiology of such lung diseases, dozens of genome-wide association studies (GWAS) have been conducted using affected patients’ DNA from blood samples (2).

Among such loci identified by GWAS, chromosome 19q13 that carries genes, including *TGFB1*, *B9D2* and *TMEM91*, has been reported to harbor multiple single-nucleotide polymorphisms (SNPs) that are linked to asthma, CF and/or COPD. For example, the SNP rs1800469 located at the intergenic non-coding region of 19q13 has been identified as linked to asthma (3–6), CF (7) and COPD (8). This SNP is traditionally recognized as C-509T located 509 bp upstream of *TGFB1* (transforming growth factor beta 1) that is involved in airway inflammation and remodeling (9–13). However, this locus is also 26 bp downstream of B9D2 (9782 bp downstream from the transcription start site) that is involved in ciliogenesis (14), which is important for airway physiological function, and 21 833 bp upstream of *TMEM91* (transmembrane protein 91), the function of which is unknown. The eQTL analysis by the GTEx Portal indicates that rs1800469 is an eQTL for multiple nearby genes, including *TGFB1*, *B9D2* and *TMEM91*, in different tissues (Supplementary Table S1; https://www.gtexportal.org/home/), suggesting that rs1800469 affects the expression of such nearby genes and in turn influences the pathogenesis of asthma, CF and COPD; however, this analysis does not provide the molecular mechanism by which rs1800469 affects the expression of the genes. A popular hypothesis to explain the mechanism is that a region harboring an SNP such as rs1800469 functions as a gene-regulatory region, thereby affecting the expression of associated genes (15). Such a hypothesis can be tested in part using epigenomic analyses, including ChIP-seq and Hi-C, led by the ENCODE project (16) (https://www.encodeproject.org), which indicates that the region harboring rs1800469 may function as a potential enhancer region (H3K27ac and H3K4me1 positive) and interact with regions harboring *TGFB1*, *B9D2* and *TMEM91*. However, the ChIP-seq analysis looking at the enhancer marks (H3K27ac and H3K4me1) does not provide information as to which expression of gene(s) (one gene or multiple genes) may be affected by the region. And the physical chromatin interaction indicated by the Hi-C analysis does not necessarily mean a functional interaction. In order to prove that the region indeed affects gene expression, a reporter assay in which a region is fused to a reporter gene such as CAT or luciferase in a plasmid (17,18) has been traditionally used; however, this assay also does not provide information as to which expression of gene(s) (one gene or multiple genes) may be affected by the region. In addition, gene regulation in a plasmid used in a reporter assay may not really reflect gene regulation in a chromatin context embedded in the actual genome. However, the recent development of genome-editing technology, including CRISPR/Cas9 and/or dCas9, now allows us to assess in a genomic context whether a region harboring a disease-associated SNP such as rs1800469 affects the expression of gene(s) (one gene or multiple genes). Such an approach using genome-editing technology has been demonstrated in multiple cell types, including human cell lines of erythroleukemia (K562), CD4^+^ T (My-La), embryonic kidney (HEK293T) and prostate cancer (LNCaP) (19–25).

In the present study, using one of the CRISPR/Cas9 genome-editing technologies (dCas9-KRAB), we determined at the genomic level the function of regions harboring SNPs linked to asthma, CF and/or COPD that are located at chromosome 19q13 (rs1800469 and rs2241712) carrying *TGFB1*, *B9D2* and *TMEM91* and chromosome 17q21 (rs4794820, rs12603332, rs7216389, rs8069176, rs8067378, rs12936231, rs9303277 and rs907091) carrying *GSDMB* and *ORMDL3* and a region harboring an SNP linked to IPF that is located at chromosome 11p15 (rs35705950) carrying *MUC5AC* and *MUC5B* as to whether such regions with the SNPs affect the expression of nearby genes in human lung epithelial cell lines and primary fibroblasts. Additionally, in order to develop a more streamlined approach (e.g. equivalent to siRNA) to assess the role of such regions with the SNPs, we tested an approach using synthetic single-guide RNA (sgRNA) to target such regions instead of constructing expression plasmids for each corresponding locus. Our present approach will enable GWAS data to be linked to molecular function beyond mere association between genomic loci and lung diseases.

## MATERIALS AND METHODS

### Vectors

CRISPR interference (CRISPRi; CRISRP/dCas9-KRAB) lentiviral vector was obtained from Addgene (pLV hU6-sgRNA hUbC-dCas9-KRAB-T2a-Puro; Plasmid #71236) deposited by Charles Gersbach (20). DNA oligos to generate each sgRNA targeting the locus (rs1800469 or rs35705950; Supplementary Table S2) were designed using CRISPOR (26) and inserted into the lentiviral vector. Lentiviruses were produced using the lentiviral vectors at the Viral Vector Core at Cincinnati Children’s Hospital Medical Center (CCHMC).

### Synthetic sgRNA

Synthetic sgRNAs targeting each locus (rs1800469, rs35705950, rs2241712, rs907091, rs9303277, rs12936231, rs8067378, rs8069176, rs7216389, rs12603332 or rs4794820; Supplementary Table S2) were designed as described above and generated using the Invitrogen custom TrueGuide gRNA (sgRNA) ordering tool (Thermo Fisher, Waltham, MA). Non-targeted gRNA (sgRNA) was used as a negative control (cat# A35526, Thermo Fisher).

### Cells

Human lung epithelial cell lines (A549 lung carcinoma cell line, H292 lung mucoepidermoid carcinoma cell line and H441 lung papillary adenocarcinoma cell line) obtained from American Type Culture Collection (ATCC, Manassas, VA) were cultured according to the methods by ATCC. Human primary lung fibroblasts were obtained from Cincinnati Fetal Center at CCHMC (IRB Study #2012-3263) and cultured in Dulbecco’s modified Eagle’s medium (cat# 11965-092, Thermo Fisher) with 10% fetal bovine serum (cat# F4135, Sigma-Aldrich, St Louis, MO) and 1% penicillin–streptomycin (cat# 15140-122, Thermo Fisher). Sanger sequencing was performed to determine SNP alleles at the DNA Sequencing and Genotyping Core at CCHMC (Supplementary Table S3). These cells were infected by lentiviruses carrying CRISPR/dCas9-KRAB as described by Thakore et al. (20) and the infected cells were selected one week after infection using puromycin (10 μg/ml for A549, H292 and H441 cells and 1 μg/ml for the fibroblasts). A549 cells expressing dCas9-KRAB were transfected with synthetic sgRNAs (50 nM at the final concentration) using Lipofectamine RNAiMAX Reagent (cat# 13778075, Thermo Fisher).

### Gene expression analyses

RNA was extracted using TRIzol reagent (cat# 15596018, Thermo Fisher). cDNA was made from the RNA using iScript™ Reverse Transcription Supermix for RT-qPCR (cat# 1708841, Bio-Rad, Hercules, CA). TaqMan gene expression analysis was performed using the cDNA to assess mRNA expression according to the manufacturer’s protocol using probes (Hs00998133 for *TGFB1*, Hs01086368 for *B9D2*, Hs04185306 for *TMEM91*, Hs00873651 for *MUC5AC*, Hs00861595 for *MUC5B*, Hs00218565 for *GSDMB*, Hs00918021 for *ORMDL3* and Hs02758991 for *GAPDH* as control from Thermo Fisher). A probe for *dCas9* was custom-designed by Thermo Fisher (F-CCTGGATTTTCTTAAGTCCGATGGA; R-GAGAGTCATCATGGATCAACTGCAT; probe-CCAACCGGAACTTC). Immunoblotting was performed to assess the protein expression of TGFB1 as described previously (27) using TGFB1 antibody (1:5000; cat# AF-246-NA, R&D Systems, Minneapolis, MN) and rabbit anti-ACTA1 as loading control (1:5000; cat# A2066, Sigma-Aldrich).

### Visualization of Hi-C and ChIP-seq data

**Figure 1.**
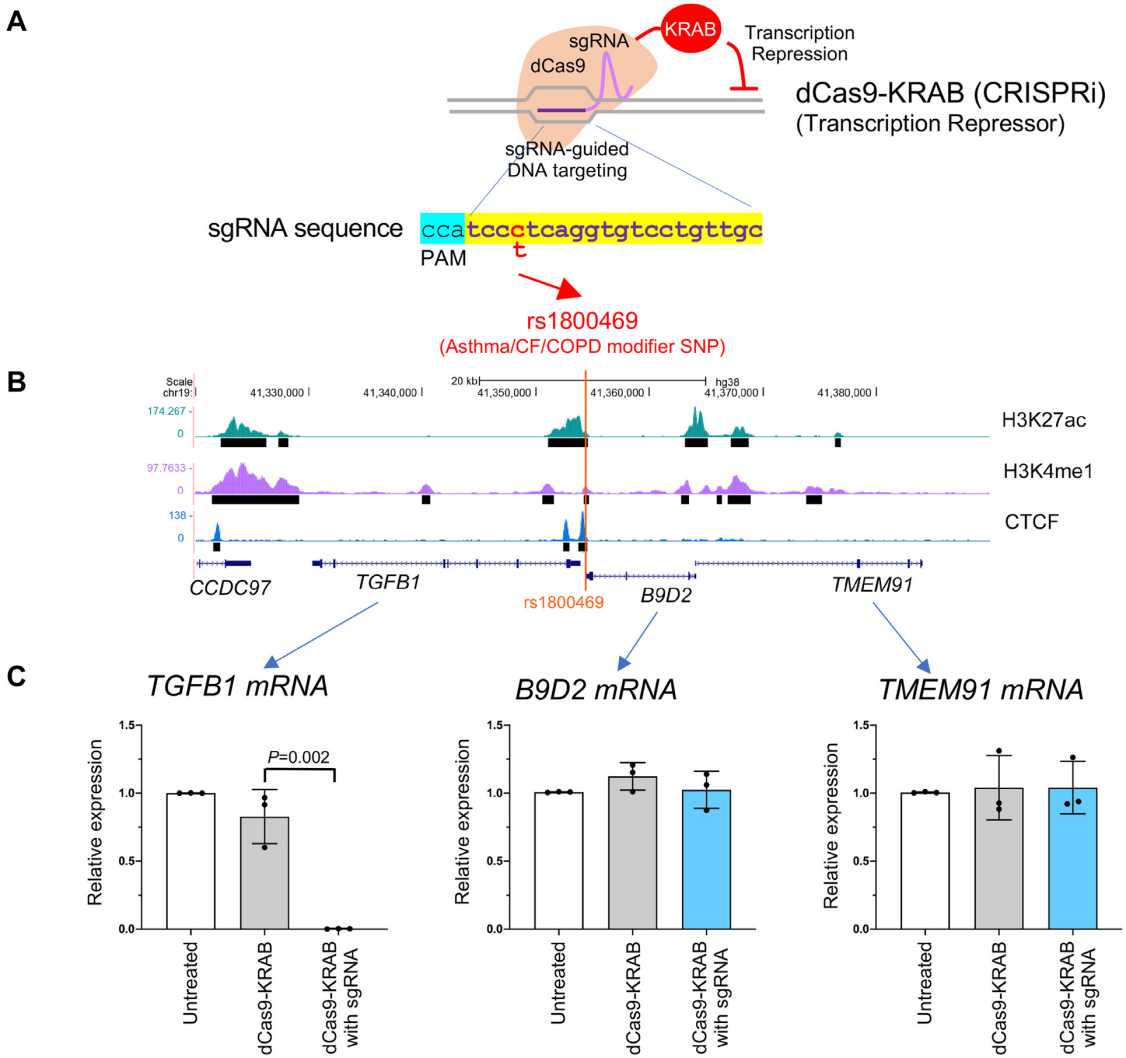
CRISPRi targeting a non-coding intergenic region that harbors SNP rs1800469 extensively represses the mRNA expression of *TGFB1* but not that of *B9D2* or *TMEM91*. (**A**) Design of the sgRNA target sequence (20-mer) containing SNP rs1800469 (asthma/CF/COPD modifier SNP), which will recruit dCas9-KRAB to the genomic matching site to repress the activity of the site as a gene-regulatory region (CRISPRi). The illustration was modified from (19). (**B**) Presumed enhancer and insulator regions at the genomic locus of *TGFB1*, *B9D2* and *TMEM91* in an A549 human lung epithelial cell line. ChIP-seq data using antibodies against H3K27ac and H3K4me1 (enhancer-specific histone marks) and CTCF (insulator-specific transcription factor) indicate that the region harboring SNP rs1800469 is in a gene-regulatory enhancer region and is blocked by an insulator region to access the genomic locus of *TGFB1*. (**C**) Lentiviral CRISPRi expressing both dCas9-KRAB and sgRNA targeting the region with SNP rs1800469 extensively repressed the expression of *TGFB1* but not that of *B9D2* or *TMEM91* compared to the control (dCas9-KRAB only). A549 cells were infected with the CRISPRi lentivirus and RNA extracted. Gene expression analysis was performed as described in the ‘Materials and Methods’ section. Data points were obtained from three independent experiments (each untreated dataset was set as 1).

Hi-C data from an A549 cell line were visualized as interaction maps and virtual 4C plots using the 3D Genome Browser (28). ChIP-seq data of H3K27ac, H3K4me1 and CTCF signals and peaks on A549 cell lines were visualized using the UCSC Genome Browser (29). The ENCODE A549 Hi-C data (ENCODE ID: ENCSR444WCZ) (30,31) processed by the 3D Genome Browser (hg38 genome assembly, ICE normalized, 10 kb resolution) were used for generating the interaction maps and virtual 4C plots. The ENCODE ChIP-seq signals and peaks (30) of H3K27ac (ENCODE ID: ENCSR783SNV), H3K4me1 (ENCODE ID: ENCSR636PIN) and CTCF (ENCODE ID: ENCSR035OXA) on an A549 cell line were downloaded from Gene Expression Omnibus (GEO; GSE91337, GSE91306 and GSE92782). Figures 1, 4, 6 and 7 and Supplementary Figures S1, S4, S6 and S7 describe signals and peaks from datasets of H3K27ac (isogenic replicate ENCLB098CDQ), H3K4me1 (isogenic replicate ENCLB391ZXW) and CTCF (isogenic replicate ENCLB141HAE). Supplementary figures describe all replicates.

### RNA-seq and ChIP-seq

RNA-seq and ChIP-seq were performed as described previously at the DNA Sequencing and Genotyping Core at CCHMC (27,32). RNA or chromatin was obtained from H441 cells infected with lentiviruses that carry CRISPR/dCas9-KRAB (control) or CRISPR/dCas9-KRAB with sgRNA targeting rs1800469. For ChIP-seq, Cas9 antibody (cat# 61757) (33) was obtained from Active Motif (Carlsbad, CA) and H3K9me3 antibody (cat# ab8898) (20) was obtained from Abcam (Cambridge, UK).

Quality assessment and pre-processing of RNA-seq reads were performed using FASTQC, Trim Galore and SAMtools. Reads were then aligned to hg38 genome using Bowtie2. Low-quality alignments and PCR duplicates were removed using SAMtools and Picard MarkDuplicates tool. Gene expression was counted using htseq-count. Differential expression analysis for each comparison was performed using Bioconductor DESeq2 package. Differential expression with at least a 2-fold change and false discovery rate <0.1 was considered significant.

Quality assessment and pre-processing of ChIP-seq reads were performed using FASTQC, Trim Galore and SAMtools. Reads were aligned to hg38 genome using Bowtie2, using the following setting: ‘--end-to-end --no-mixed --no-discordant --minins 100 --maxins 1000 -x hg38’ as described in (33). Low-quality alignments and duplicate reads were removed using SAMtools and Picard MarkDuplicates tool. MACS2 software (version 2.1.0) was used for ChIP-seq peak calling. dCas9 peaks were called using ‘macs2 callpeak -t dCas9_ab.bam -c input.bam -f BAM -g hs -B --keep-dup 1 -q 0.01’ and H3K9me3 peaks were called with ‘macs2 callpeak -t H3K9me3_ab.bam -c input.bam -f BAM -g hs -B --keep-dup 1 --broad --broad-cutoff 0.1 -p 0.05’. Homer mergePeaks tool (version 4.10) was used with parameter ‘-d 100’ to separate peaks from different condition–antibody–replicate combinations into unique and overlapping sets and compared with off-target regions predicted by CRISPOR. Homer annotatePeaks.pl tool (version 4.10) was used to annotate the ‘nearest gene’ information of peak regions.

### Statistics

Real-time qPCR results were normalized against the untreated samples in three or more independent biological replicates. Results are expressed as the mean ± SD of the replicates for each group. Statistical relevance was determined using Student’s *t*-test on treated versus control treated relative expression with a minimum *P*-value <0.05. GraphPad Prism 8 was used for graphing and statistical analysis.

## RESULTS

### Non-coding region harboring SNP rs1800469 functions as an enhancer for the expression of a single gene

We hypothesized that the intergenic non-coding region harboring SNP rs1800469 functions as a gene-regulatory region; thus, the SNP influences the gene-regulatory activity of the region to control genes that affect the pathogenesis of asthma, CF and COPD. In order to identify such genes controlled by the region, we used the CRISPRi (CRISPR/dCas9-KRAB) system, a modified CRISPR/Cas9 genome-editing technology that uses a deactivated Cas9 fused to the repressor KRAB, to repress the transcription of genes by binding to gene-regulatory regions of genomic DNA selected by sgRNA target sequences (20-mer) without making DNA double-stranded breaks (19,20). We were able to find an sgRNA target sequence that contains the SNP within the 20-mer sequence, which in theory assures the direct binding of dCas9-KRAB to the region with the SNP (Figure 1A). We subsequently generated a CRISPRi lentivirus carrying dCas9-KRAB and sgRNA targeting the region. Then, we infected A549 lung epithelial cells with the CRISPRi lentivirus and assessed the expression of three genes (*TGFB1*, *B9D2* and *TMEM91*) that are located most closely to the SNP. Although the publicly available bioinformatical datasets (Hi-C and ChIP-seq) indicate that the region interacts with other genomic regions harboring the loci of *TGFB1*, *B9D2* and *TMEM91* (Supplementary Figure S1A and B) and resides on an enhancer region marked by enhancer-specific histone modifications (H3K27ac and H3K4me1; Figure 1B and Supplementary Figure S1C and D), CRISPRi targeting the region significantly repressed the expression of *TGFB1* (99.6%; *P* = 0.002) but not that of *B9D2* (8.8%; *P* = 0.37) or *TMEM91* (0%; *P* = 0.999) compared to the control CRISPRi (Figure 1C and Supplementary Figure S2). These results indicate that the region harboring SNP rs1800469 functions as an enhancer region to regulate the expression of a single gene (*TGFB1*) but not multiple genes, which in turn influences the pathogenesis of asthma, CF and COPD. This result also indicates that CRISPRi (but not Hi-C, which provides genomic region–region interaction information, or ChIP-seq, which provides presumed gene-regulatory region information) can specify the expression of which gene(s) is influenced by such gene-regulatory regions.

### Function of non-coding enhancer harboring SNP rs1800469 was cell-type independent

**Figure 2.**
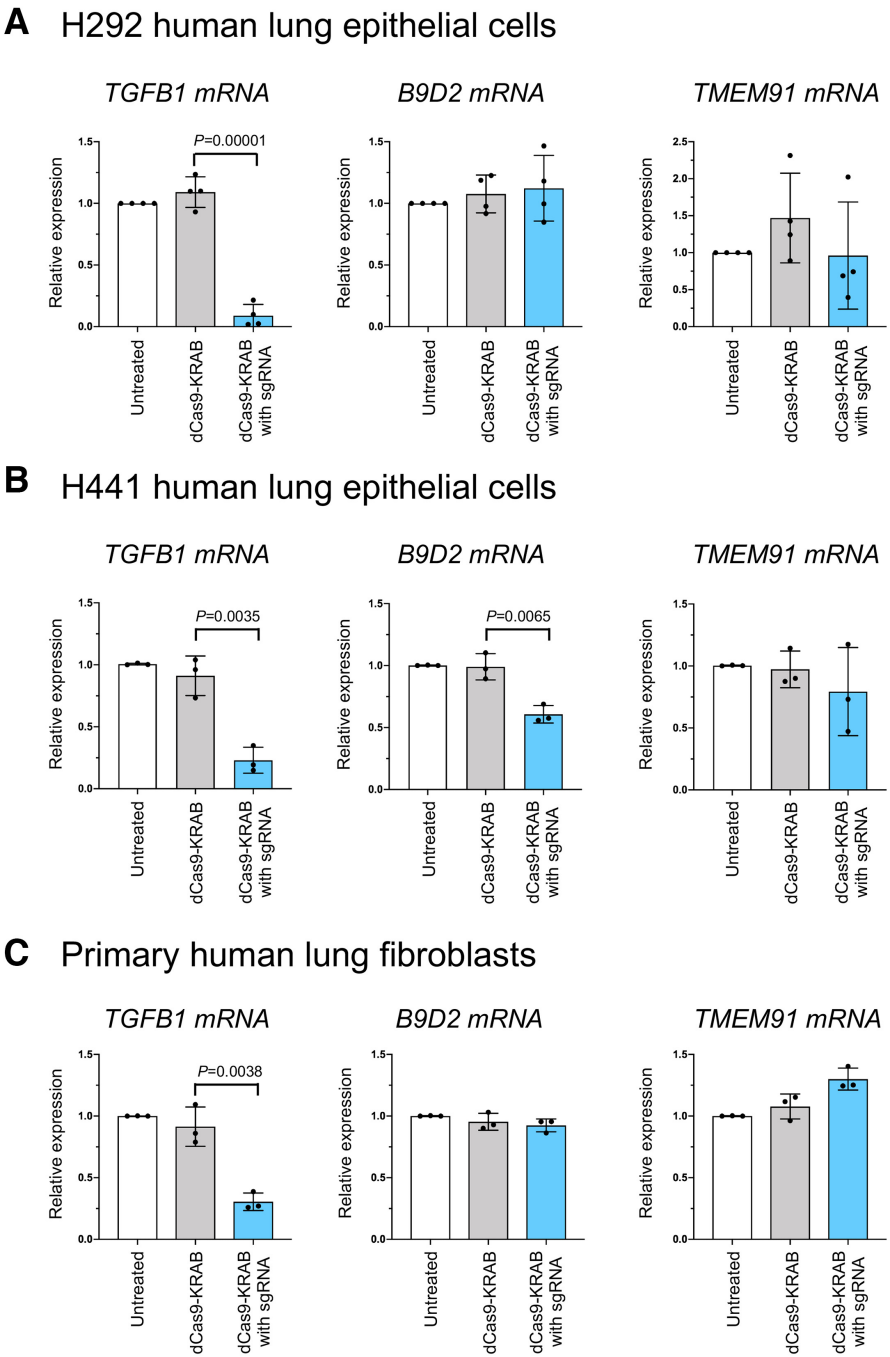
CRISPRi targeting the region with SNP rs1800469 inhibited the expression of *TGFB1* in different cell types, including human primary lung fibroblasts. (**A**) The lentiviral CRISPRi described in Figure 1 significantly repressed the expression of *TGFB1* but not that of *B9D2* or *TMEM91* compared to the control (dCas9-KRAB only) in an H292 lung epithelial cell line. Data points were obtained from four independent experiments (each untreated dataset was set as 1). (**B**) The lentiviral CRISPRi described in Figure 1 also significantly repressed the expression of *TGFB1* and *B9D2* compared to the control (dCas9-KRAB only) in an H441 lung epithelial cell line; however, the repressive effect on *B9D2* was <2-fold. Data points were obtained from three independent experiments (each untreated dataset was set as 1). (**C**) The lentiviral CRISPRi described in Figure 1 also significantly repressed the expression of *TGFB1* compared to the control (dCas9-KRAB only) in human primary lung fibroblasts. Data points were obtained from three independent experiments (each untreated dataset was set as 1).

We further assessed the role of the region harboring SNP rs1800469 in additional lung epithelial cell lines (H292 and H441). In H292 and H441 cells, the CRISPRi targeting this region significantly repressed the expression of *TGFB1* >2-fold (log_2_ fold change ≥1) but not that of *B9D2* and *TMEM91* (Figure 2A and B), which is consistent with the result using A549 cells (Figure 1). Since *TGFB1* is expressed not only in lung epithelial cells but also in non-epithelial cells, we also used the CRISPRi to determine whether the region influences the expression of *TGFB1* in primary human lung fibroblasts. Consistent with the results using lung epithelial cell lines (A549, H292 and H441), the CRISPRi significantly repressed the expression of *TGFB1* (>2-fold; log_2_ fold change ≥1) but not that of *B9D2* or *TMEM91* in the fibroblasts (Figure 2C and Supplementary Table S4; of note, the expression levels of the genes are comparable in the four cell types). Although A549 and H292 cells carry the C allele and H441 cells and the fibroblasts carry the T allele (Supplementary Table S3), the repressive effect by the CRISPRi was not influenced (Figures 1 and 2) probably because such single-nucleotide mismatches are normally tolerated by the CRISPR system as reported previously (34). These results indicate that the region harboring SNP rs1800469 functions as an enhancer region to control the expression of a single gene only (*TGFB1*) regardless of cell type.

### CRISPRi was tightly controlled in an sgRNA-specific fashion

**Figure 3.**
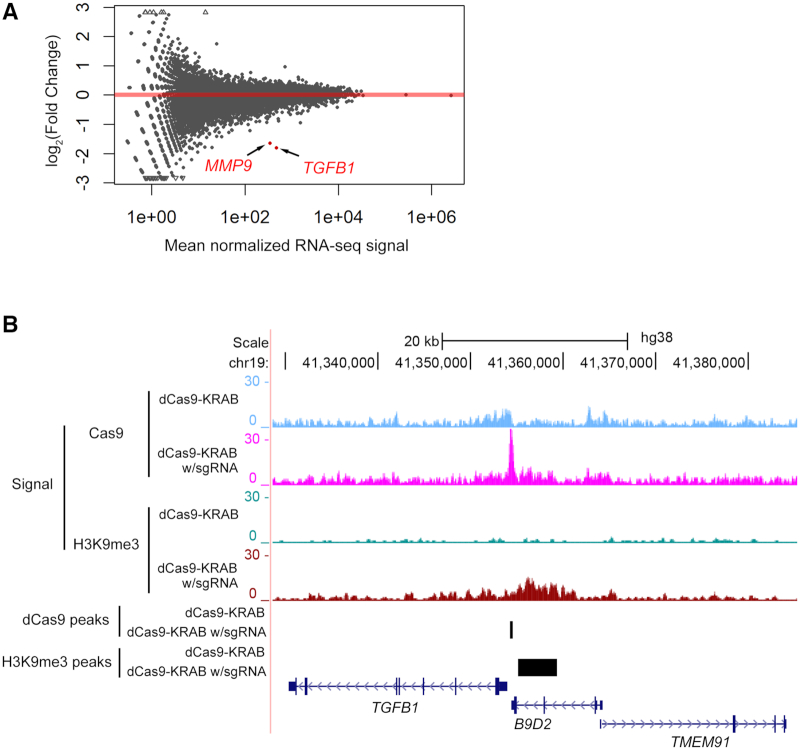
RNA-seq and ChIP-seq indicate that the CRISPRi targeting rs1800469 regulates the expression of *TGFB1* and tightly associates with a region specified by the sgRNA target sequence. (**A**) *TGFB1* was most highly repressed by the CRISPRi targeting rs1800469. RNA-seq was performed using RNAs from H441 cells infected with the lentiviral CRISPRi expressing both dCas9-KRAB and sgRNA targeting the region with SNP rs1800469 and the control (dCas9-KRAB only). Three replicates were used for each group. Red points represent genes with log_2_ fold change ≥1 or ≤−1; *p*_adj_ <0.1. (**B**) ChIP-seq data using H441 cells indicates that the CRISPRi targeting rs1800469 bound to a region that matches to our gRNA target sequence. A histone repressive mark (H3k9me3) was adjacently associated with the binding of the CRISPRi targeting rs1800469. The analyses were performed twice independently (see Supplementary Figure S3).

Since the eQTL analysis by the GTEx Portal indicates that rs1800469 is an eQTL for multiple nearby genes in addition to *TGFB1*, *B9D2* and *TMEM91* (Supplementary Table S1; of note, multiple tissues are used for the eQTL analysis by the GTEx Portal, https://www.gtexportal.org/home/), we sought to determine which genes might be regulated by the CRISPRi using RNA-seq in an unbiased fashion using H441 cells. As shown in Figure 3A and Supplementary Table S5, the CRISPRi significantly repressed *TGFB1* and then *MMP9* but not the other genes (log_2_ fold change ≥1 or ≤−1; *p*_adj_ < 0.1), including the genes identified by the eQTL analysis for rs1800469 (Supplementary Table S1). These data indicate that the CRISPRi tightly regulates the expression of *TGFB1* in an sgRNA target sequence-specific manner and suggest a limitation of eQTL analysis to precisely determine whether a region harboring an SNP is a gene-regulatory region (35). The repressed expression of *MMP9* might be secondary to the CRISPRi-mediated repression of *TGFB1* but not an off-target effect since MMP9 is known to be a TGFB1 downstream gene (36–38) and the sgRNA design tool CRISPOR did not detect the locus of *MMP9* (as expected, CRISPOR detected the region harboring rs1800469 as ‘intergenic: TMEM91-B9D2’, which we used as an sgRNA target sequence; Supplementary Table S6). However, in order to assess the potential off-target effect, we performed ChIP-seq using antibodies against Cas9 (33) and H3K9me3 (a histone repressive mark added by KRAB) (20) and sought to determine where the CRISPRi is located and whether nearby chromatin is in a repressed state. As shown in Figure 3B and Supplementary Figure S3, Cas9 in the CRISPRi was located at the region harboring rs1800469 and H3K9me3 was adjacently associated with the region, indicating that the CRISPRi is specifically recruited based on the sgRNA target sequence and suppressed the expression of *TGFB1*. Such specific recruitment of Cas9 was not seen in other loci in the genome, including the locus of *MMP9* (Supplementary Table S7).

### Non-coding region harboring SNP rs35705950 functions as an enhancer for the expression of multiple genes

**Figure 4.**
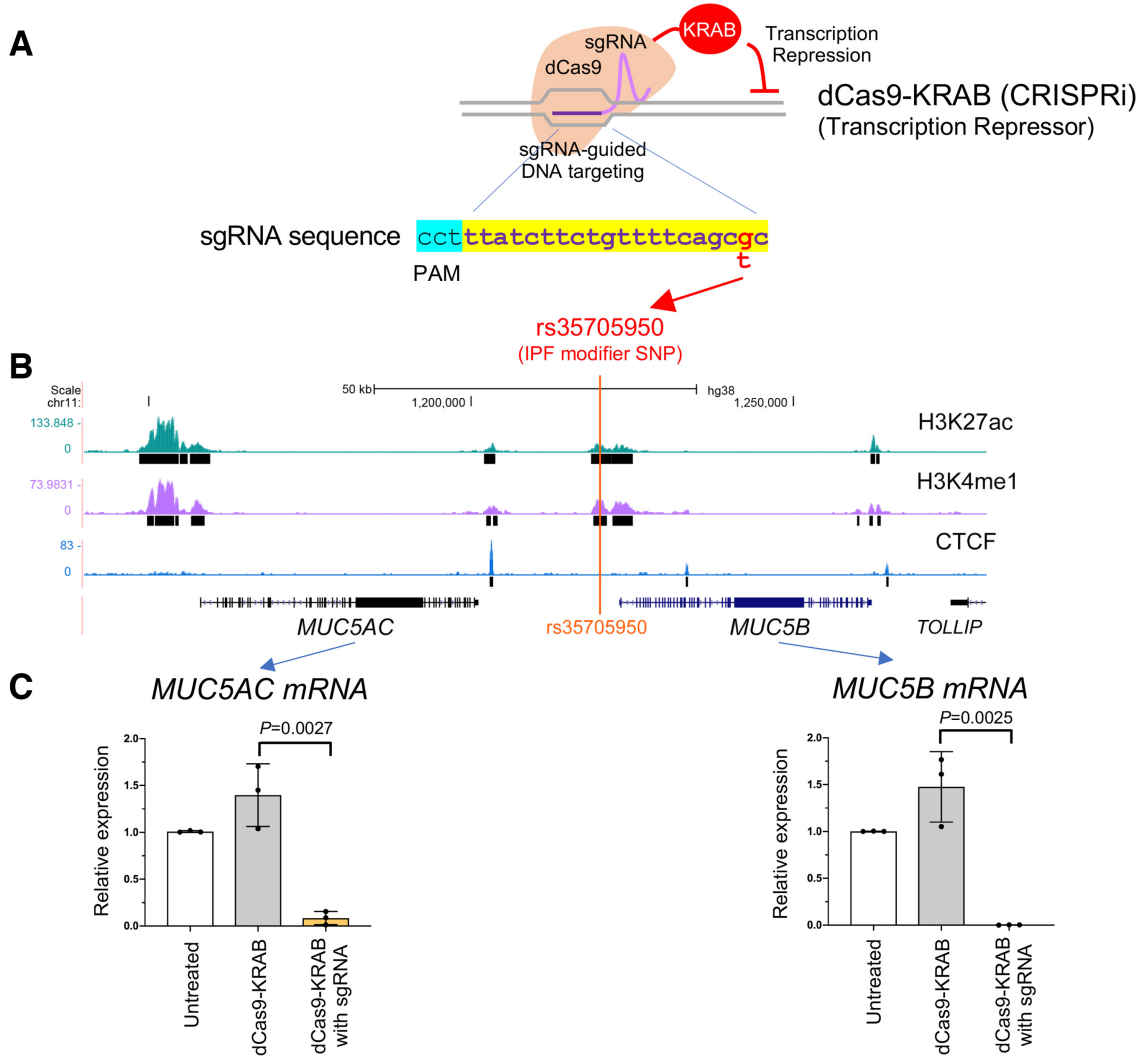
CRISPRi targeting a non-coding intergenic region that harbors SNP rs35705950 represses the mRNA expression of both *MUC5AC* and *MUC5B*. (**A**) Design of sgRNA target sequence (20-mer) containing SNP rs35705950 (IPF modifier SNP), which will recruit dCas9-KRAB to the genomic site as described in Figure 1 (CRISPRi). The illustration was modified as described in Figure 1A. (**B**) Presumed enhancer and insulator regions at the genomic locus of *MUC5AC* and *MUC5B* in A549 cells. ChIP-seq data using antibodies as described in Figure 1 indicate that the region harboring SNP rs35705950 is in a gene-regulatory enhancer region and is blocked by an insulator region to access the genomic locus of *MUC5AC*. (**C**) Lentiviral CRISPRi expressing both dCas9-KRAB and sgRNA targeting the region with SNP rs35705950 significantly repressed the expression of both *MUC5AC* and *MUC5B* compared to the control (dCas9-KRAB only) in A549 cells. Data points were obtained from three independent experiments (each untreated dataset was set as 1).

We previously identified a functional enhancer region at an intergenic non-coding region of chromosome 11p15 (between *MUC5AC* and *MUC5B*) using CRISPR/Cas9 (but not CRISPRi) that influences the mRNA expression of *MUC5B* (32). Notably, this region harbors the SNP rs35705950 that is associated with IPF, supposedly inducing the expression of MUC5B and in turn promoting IPF pathogenesis (39). Our previous approach to determine whether this region functions as an enhancer region was to make two DNA double-stranded breaks at both ends of the region using CRISPR/Cas9 (but not CRISPRi) and establish cell clones that lack the region (746 bp deletion), which is a time-consuming process (32). Here, we sought a more convenient approach using CRISPRi to determine whether the region harboring SNP rs35705950 is a functional enhancer region. We were able to find an sgRNA target sequence harboring SNP rs35705950 within a 20-mer sequence and then generated lentivirus expressing CRISPRi (dCas9-KRAB) and the sgRNA (Figure 4A). The Hi-C data using A549 cells indicate that this region associates with both the *MUC5AC* genomic locus and the *MUC5B* genomic locus (Supplementary Figure S4A and B) and the ChIP-seq data using A549 cells indicate that the region resides on a presumed enhancer region (H3K27ac and H3K4me1 positive; Figure 4B and Supplementary Figure S4C and D); however, these datasets do not establish whether the expression of each gene is influenced by the region. We therefore used the CRISPRi lentivirus targeting this region to determine whether the region influences the mRNA expression of *MUC5AC* and/or *MUC5B* in A549 cells. Notably, CRISPRi targeting the region repressed the expression of both *MUC5AC* (93.9%; *P* = 0.0027) and *MUC5B* (99.9%; *P* = 0.0025), indicating that the region harboring SNP rs35705950 functions as an enhancer for multiple gene expression (Figure 4C). These results also indicate that CRISPRi is a convenient approach to determine whether genomic regions harboring chronic lung disease-associated SNPs function as gene-regulatory regions.

### Synthetic sgRNA as a streamlined tool to determine the function of non-coding regions harboring disease-associated SNPs

**Figure 5.**
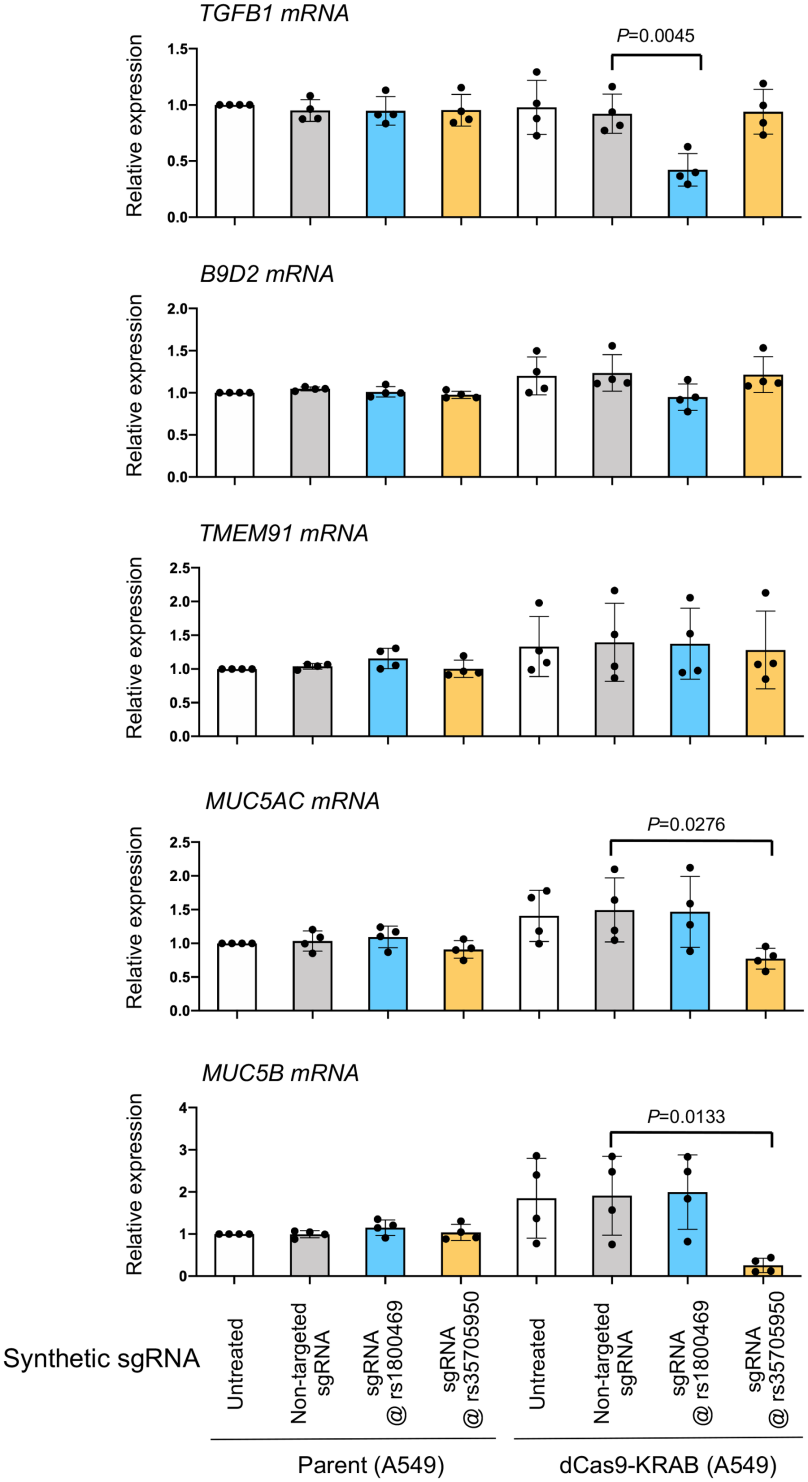
Synthetic sgRNA targeting a non-coding intergenic region that harbors SNP rs1800469 (asthma/CF/COPD modifier SNP) or rs35705950 (IPF modifier SNP) repressed the expression of *TGFB1* or *MUC5AC*/*MUC5B*, respectively, but not that of other genes. A549 cells stably expressing dCas9-KRAB generated by lentivirus infection were transfected with synthetic sgRNAs targeting the genomic region (20-mer; see Figures 1A and 4A) that harbors SNP rs1800469 or SNP rs35705950. Two days after transfection, RNA was extracted from the transfected cells and gene expression analysis was performed as described in the ‘Materials and Methods’ section. Shown are data from four independent biological replicates (each untreated dataset was set as 1).

Although the CRISPRi approach described above using a lentivirus that expresses dCas9-KRAB and sgRNA is a convenient approach to determine gene-regulatory regions (Figures 1–4) compared to the deletion approach using CRISPR/Cas9 (32), it requires a custom vector construction process to insert oligos matching sgRNA sequences into a lentiviral vector. In order to bypass the vector construction process, we assessed whether transfecting synthetic sgRNAs into cells stably expressing dCas9-KRAB can replace the approach using individual lentivirus constructions. We synthesized sgRNAs targeting the two SNPs (rs1800469 or rs35705950) and transfected them into A549 cells that stably express dCas9-KRAB. The synthetic sgRNA targeting rs1800469 (same sequence as Figure 1A) repressed the expression of *TGFB1* (54.2%; *P* = 0.0045) but no other genes (*B9D2*, *TMEM91*, *MUC5AC* and *MUC5B*; <23.2%; *P* > 0.05) and the synthetic sgRNA targeting rs35705950 (same sequence as Figure 4A) repressed the expression of *MUC5AC* (48.3%; *P* = 0.0276) and *MUC5B* (86.6%; *P* = 0.0133) but no other genes (*TGFB1*, *B9D2* and *TMEM91*; <8.1%; *P* > 0.05; Figure 5 and Supplementary Figure S5), the results of which are consistent with the approach using individual lentivirus construction (Figures 1-4). The approach using synthetic sgRNA in cells that stably express dCas9-KRAB is a further streamlined approach to quickly determine the gene-regulatory role(s) of the regions harboring the SNPs.

### Functions of genomic regions harboring SNPs located at chromosomes 19q13 and 17q21 that are linked to asthma and COPD were quickly determined using synthetic sgRNA

**Figure 6.**
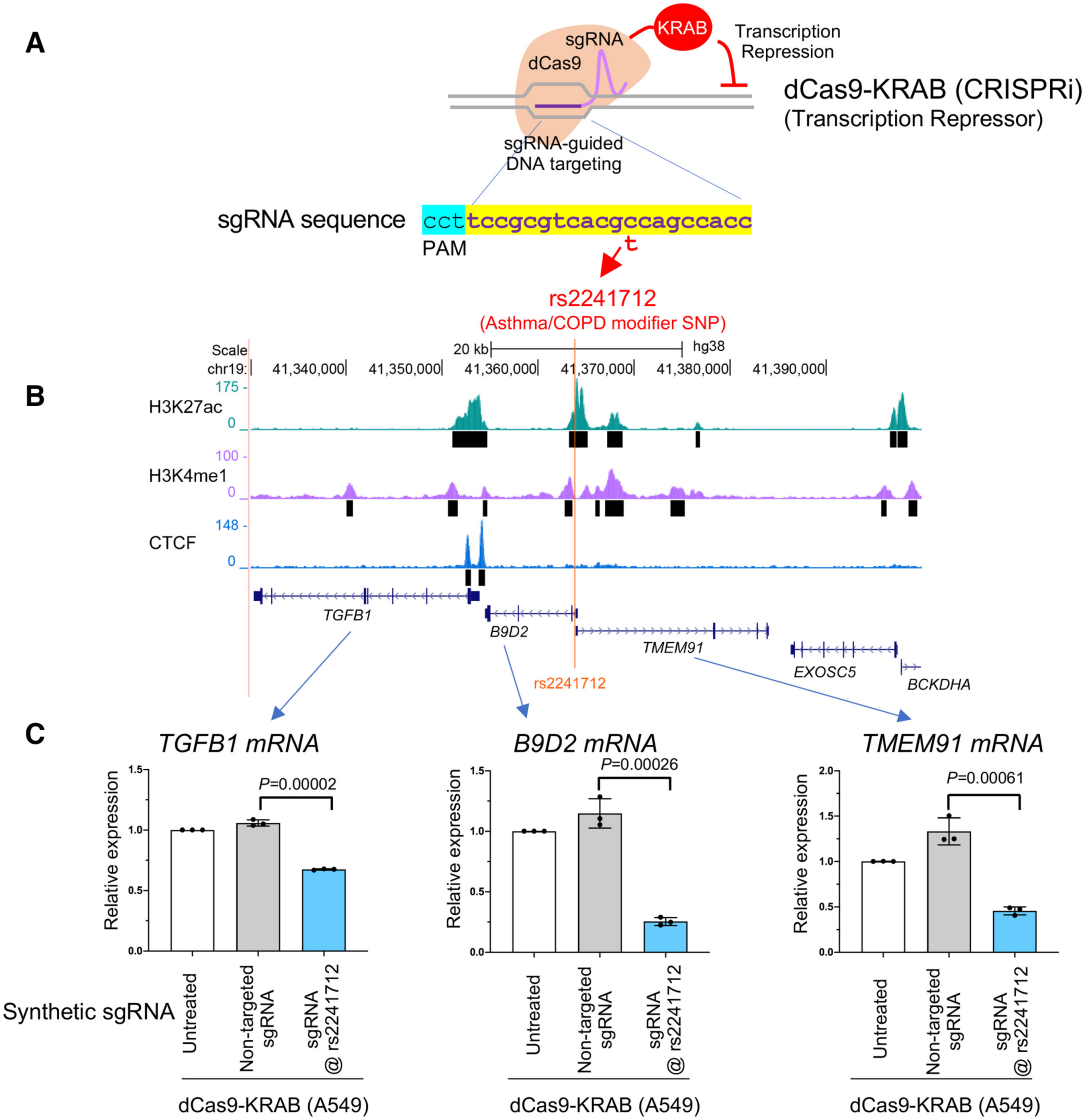
Synthetic sgRNA targeting a non-coding intronic region that harbors SNP rs2241712 (asthma/COPD modifier SNP) repressed the expression of *TGFB1*, *B9D2* and *TMEM91*. (**A**) Design of sgRNA target sequence (20-mer) containing SNP rs2241712 (asthma/COPD modifier SNP), which will recruit dCas9-KRAB to the genomic site as described in Figure 1 (CRISPRi). The illustration was modified as described in Figure 1A. (**B**) Presumed enhancer and insulator regions at the genomic locus of *TGFB1*, *B9D2* and *TMEM91* in A549 cells. ChIP-seq data using antibodies as described in Figure 1 indicate that the region harboring SNP rs2241712 is on a gene-regulatory enhancer region (H3K27ac) and is blocked by an insulator region to access the genomic locus of *TGFB1*. (**C**) A549 cells stably expressing dCas9-KRAB were transfected with synthetic sgRNAs targeting the genomic region that harbors SNP rs2241712 as described in Figure 5. Gene expression analysis was performed as described in Figure 5. Shown are data from three independent biological replicates (each untreated dataset was set as 1).

Chromosome 19q13 harbors another SNP (rs2241712) in addition to rs1800469 that is linked to asthma (6) and COPD (8). In order to determine the function of the region harboring rs2241712, we used the synthetic sgRNA approach described in Figure 5 and sought to determine whether the region regulates the expression of *TGFB1*, *B9D2* and *TMEM91* (Figure 6A and Supplementary Figure S6). Rs2241712 is located at the first intron of *B9D2* (2 kb upstream region of *TMEM91*) and the Hi-C and ChIP-seq data indicate that this region interacts with other genomic regions harboring the loci of *TGFB1*, *B9D2* and *TMEM91* (Supplementary Figure S6A and B) and presumably functions as an enhancer (H3K27ac positive; Figure 6B and Supplementary Figure S6C). The eQTL data indicate that this SNP is an eQTL for multiple genes, including *TGFB1*, *B9D2* and *TMEM91*. Notably, CRISPRi with synthetic sgRNA targeting the region that harbors rs2241712 repressed the expression of *TGFB1* (36.2%; *P* = 0.00002), *B9D2* (77.8%; *P* = 0.00026) and *TMEM91* (65.7%; *P* = 0.00061), indicating that this region functions as an enhancer for the expression of multiple genes (Figure 6C) in contrast to the region harboring rs1800469 at the same chromosome 19q13 that functions as an enhancer for the expression of a single gene (Figures 1 and 5).

**Figure 7.**
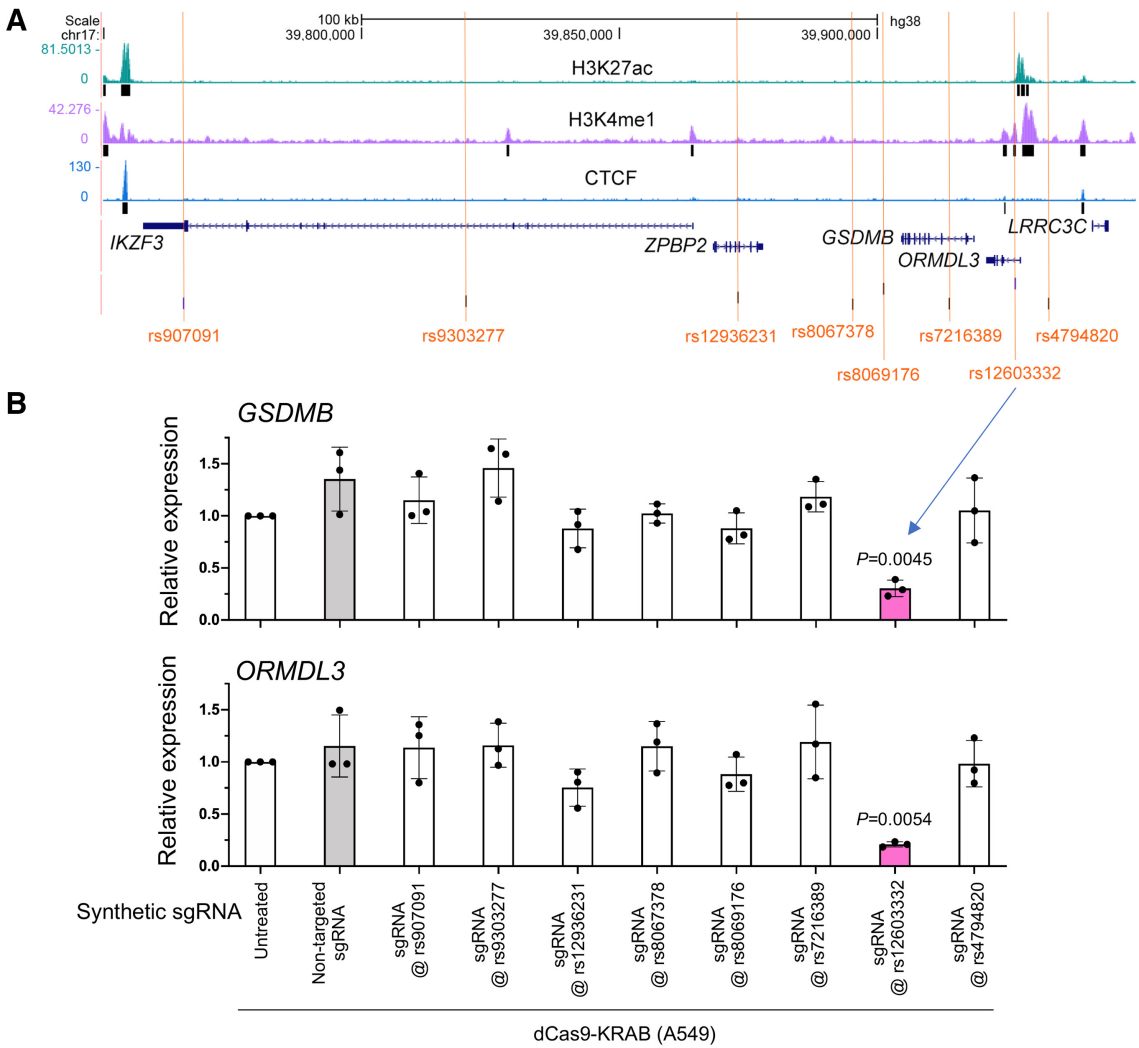
Synthetic sgRNA targeting a non-coding intronic region that harbors SNP rs12603332 but not the other SNPs (asthma modifier SNPs) located at chromosome 17q21 repressed the expression of both *GSDMB* and *ORMDL3*. (**A**) Presumed enhancer and insulator regions at the genomic locus of *GSDMB* and *ORMDL3* in A549 cells. ChIP-seq data using antibodies as described in Figure 1 indicate that the region harboring only SNP rs12603332 is on a gene-regulatory enhancer region (H3K4me1). (**B**) A549 cells stably expressing dCas9-KRAB were transfected with synthetic sgRNAs targeting the genomic regions that harbor asthma modifier SNPs as described in Figure 5. Gene expression analysis was performed as described in Figure 5. Shown are data from three independent biological replicates (each untreated dataset was set as 1). *P*-values were obtained by comparing the values of non-targeted sgRNA with those of sgRNA@rs12603332.

Since this methodology using synthetic sgRNA is basically the same as the gene-knockdown approach using siRNA, it enables us to quickly determine the functions of regions harboring multiple SNPs at the same chromosome locus simultaneously. The chromosome 17q21 locus that carries *GSDMB* and *ORMDL3* harbors more than a dozen SNPs linked to asthma (40–44). We chose eight SNPs (rs907091, rs9303277, rs12936231, rs8067378, rs8069176, rs7216389, rs12603332 and rs4794820) from those that are located at non-coding regions as well as cited as asthma loci by SNPedia (45) and sought the function of the regions that harbor the SNPs using eQTL, Hi-C, ChIP-seq and synthetic sgRNA-mediated CRISPRi approaches. The eQTL data indicate that all of the eight SNPs are eQTL for multiple genes, including *GSDMB* and *ORMDL3* that are expressed in lung epithelial cells (https://research.cchmc.org/pbge/lunggens/mainportal.html) and influence asthma pathogenesis (Supplementary Table S1) (46,47). The Hi-C data indicate that there are physical chromatin interactions in the chromosome 17q21 locus harboring the eight SNPs (Supplementary Figure S7A); however, the ChIP-seq data indicate that only rs12603332 was on the region labeled by an enhancer mark (H3K4me1; Figure 7A and Supplementary Figure S7B). Importantly, the synthetic sgRNA-mediated CRISPRi (Supplementary Table S2) indicated that only a region harboring rs12603332, but not the other seven SNP-containing regions, acted significantly as an enhancer region to control the expression of multiple genes—*GSDMB* (77.5% repression; *P* = 0.0045) and *ORMDL3* (81.9% repression; *P* = 0.0054) (Figure 7B). These results indicate that the synthetic sgRNA-mediated CRISPRi analysis should be included as a relatively simple functional assay to complement eQTL, Hi-C and ChIP-seq analyses to precisely determine the gene-regulatory role of a region that harbors lung disease-associated SNPs.

## DISCUSSION

In the present study, using CRISPRi, we demonstrated that the non-coding regions that harbor SNPs associated with asthma, CF, COPD and IPF function as gene-regulatory enhancer regions for the expression of single or multiple genes. The CRISPRi approach enabled us to assess the endogenous mRNA expression of genes relevant to such chronic lung diseases, which was not feasible before the genome-editing era.

In order to identify genetic variants that associate with chronic lung diseases, dozens of GWAS have been conducted. Notably, the majority of SNPs that are associated with such diseases are identified in non-coding regions, including intergenic and intronic regions. Such studies, in support with the eQTL analyses, often conclude with an implication that an SNP affects the expression of nearby genes (e.g. Supplementary Table S1), thereby influencing the pathogenesis of the lung diseases associated with the SNP. Before the genome-editing era, in order to understand the molecular function of such SNP harboring regions, electrophoretic mobility shift assay (EMSA, also known as gel-shift assay) and reporter assays have been used. EMSA using protein extracts (including DNA-binding proteins) and DNA oligonucleotides enables one to determine whether protein extracts differentially bind to the DNA oligonucleotides with or without an SNP; however, it does not determine whether the SNP influences the expression of disease-relevant genes that are associated with the SNP. In the reporter assay, a DNA fragment containing a region with a disease-associated SNP is fused to a reporter gene (e.g. luciferase) in a plasmid, which enables one to assess whether the region that functions as a gene-regulatory region is influenced by the SNP; however, the assay determines only whether the region influences the expression of luciferase but not whether the region influences the mRNA expression of specific genes at a genomic level. The reporter assay also has a limitation in determining intrinsic gene regulation since it is conducted using a bacterial plasmid without intact chromatin in mammalian cells that are composed of not only DNA but also histones and other DNA-binding proteins.

**Figure 8.**
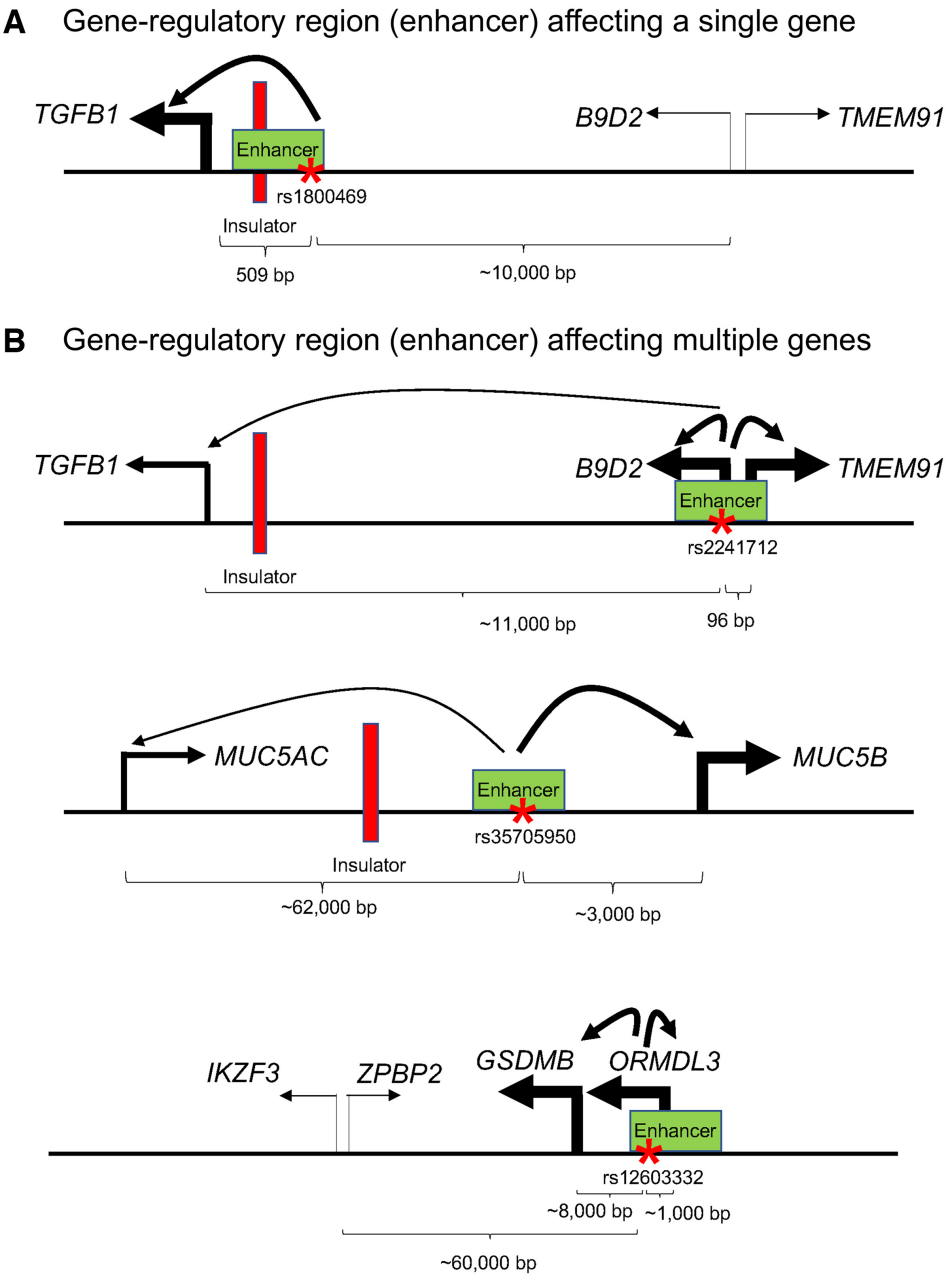
Summary of gene-regulatory mechanisms by which non-coding regions harboring lung disease-associated SNPs influence the expression of genes that are involved in disease pathogenesis. (**A**) The genomic region with the SNP rs1800469 affects the expression of a single gene *TGFB1* but not that of other genes *B9D2* or *TMEM91* despite an insulator region located between the genomic loci of *TGFB1* and *B9D2*. (**B**) The genomic regions with SNPs rs2241712, rs35705950 and rs12603332 affected the expression of multiple genes regardless of insulator regions.

Recent advances in the understanding of epigenetics involved in chromatin biology along with cost-effective next-generation sequencing have led to the determination of which genomic regions composed of chromatin interact with other genomic regions. This advance was achieved by chromosome conformation capture (only interaction of a targeted region is assessed) or Hi-C (all interactions of genomic regions are assessed by sequencing) (48). The datasets obtained from these assays provide potential genes influenced by a region that harbors a disease-associated SNP by assuming that their physical genomic interaction may contribute to their gene regulation. Additionally, such technological advances (e.g. ChIP-seq) enable us to assess potential gene-regulatory regions (e.g. enhancer or insulator) marked by specific histone modifications and DNA-binding proteins (49). Thus, if a disease-associated SNP resides in a potential enhancer region, it is presumed that the SNP may be involved in gene expression. In order to address the limitations of the EMSA and reporter assays, these recent analyses using Hi-C and ChIP-seq have been used to determine the role of disease-associated SNPs (50). We incorporated the publicly available datasets obtained using Hi-C and ChIP-seq to understand the role of the studied SNPs, which suggested that the region harboring the SNPs would interact with genomic regions containing multiple gene loci and function as enhancer gene-regulatory regions (Figures 1, 4, 6 and 7 and Supplementary Figures S1, S4, S6 and S7). Notably, the dataset obtained by CTCF ChIP-seq analysis indicated that insulator regions are located between the loci of *TGFB1* and *B9D2* and between the loci of *MUC5AC* and *MUC5B* (Figures 1B, 4B and 8 and Supplementary Figures S1C and D and S4C and D), suggesting that the region with SNP rs1800469 regulates the expression of *B9D2* and *TMEM91* (but not that of *TGFB1*) and the region with SNP rs35705950 regulates the expression of *MUC5B* (but not that of *MUC5AC*). Importantly, our results using CRISPRi indicated that the region with SNP rs1800469 regulated the expression of *TGFB1* (but not that of *B9D2* or *TMEM91*; Figure 1C), which indicates that such an enhancer region may function as a gene-regulatory region for the expression of a single gene only regardless of the physical genomic (chromatin) interaction (Supplementary Figure S1A and B) or the presence of the insulator region bound by CTCF (Figure 1B and 8 and Supplementary Figures S1C and D). In contrast, the other CRISPRi result indicated that the region with SNP rs35705950 regulated the expression of both *MUC5AC* and *MUC5B* (Figure 4C), indicating that the region functions as a gene-regulatory region for the expression of multiple genes consistent with the physical genomic (chromatin) interaction (Supplementary Figure S4A and B); however, the insulator region bound by CTCF between *MUC5AC* and *MUC5B* did not block the activity of the gene-regulatory region (Figure 4B and Supplementary Figure S4C), thereby the expression of *MUC5AC* was affected (Figures 4C and 8). Our results are consistent with other reports that CTCF binding sites do not function as critical insulators in some contexts (51,52). These results indicate that although the bioinformatical datasets obtained by Hi-C and ChIP-seq provide potential functions of genomic regions harboring disease-associated SNPs, the actual functions of the regions have to be validated by a genome-editing approach, such as CRISPRi.

The invention of the siRNA-mediated gene-knockdown approach combined with cell transfection methods accelerated the understanding of the role of genes due to the convenience compared to the use of custom plasmids that produce shRNA, an unprocessed form of siRNA (53). In the present study, we adopted the CRISPRi approach to determine whether synthetic sgRNA functions equivalently to sgRNA produced from a CRISPR plasmid (54). Notably, the results obtained using synthetic sgRNA (Figure 5) were consistent with those using a lentiviral vector (plasmid) expressing sgRNAs (Figures 1 and 4). The advantage of using synthetic sgRNA is its convenience in simply transfecting the synthetic sgRNA into cells that stably express dCas9-KRAB without the need of plasmids. Additionally, the use of synthetic sgRNA may be superior to the approach using a lentiviral vector producing both dCas9-KRAB and sgRNA, since the latter has difficulty in generating the same amount of *dCas9-KRAB* as that produced from the control lentivirus-infected cells (only dCas9-KRAB; see Supplementary Figure S8) due to the nature of the lentivirus infection scheme (random insertion).

Here, using CRISPRi (CRISPR/dCas9-KRAB), we determined the functional role of non-coding regions harboring SNPs associated with asthma, CF, COPD and IPF. Our results indicate that the datasets obtained by eQTL, Hi-C and ChIP-seq analyses have limitations in elucidating the role of such SNP-containing regions but are useful as a preliminary assessment. Any such regions identified by eQTL, Hi-C and ChIP-seq should be further analyzed by a functional approach using genome-editing technologies such as CRISPRi. With the development of more human cell lines, including primary airway cells (e.g. SCGB1A1-positive cells) (55) at air–liquid interface and/or in a novel organoid culture system (56) with stable expression of dCas9-KRAB or other modified Cas9, the functional roles of genomic regions harboring other SNPs in such chronic lung diseases will be further revealed, which may improve predictions of prognosis and therapeutic strategies. This approach can be applied to other SNPs associated with additional lung diseases, including acute respiratory distress syndrome, pneumonia, respiratory infection (e.g. COVID-19 and influenza), lung cancer and other diseases in general.

## DATA AVAILABILITY

The RNA-seq and ChIP-seq data have been deposited in the GEO database under the accession number GSE145530.

## Supplementary Material

lqaa036_Supplemental_Files
