# Supplementary material for: CRISPRi-mediated functional analysis of lung disease-associated loci at non-coding regions
Source: NAR Genom Bioinform. 2020 May 25;2(2):lqaa036. doi: 10.1093/nargab/lqaa036 (PMC7252574; doi:10.1093/nargab/lqaa036)
Supplement: lqaa036_Supplemental_Files [file lqaa036_supplemental_files.zip › NARGB Sup figures _041920.pdf]

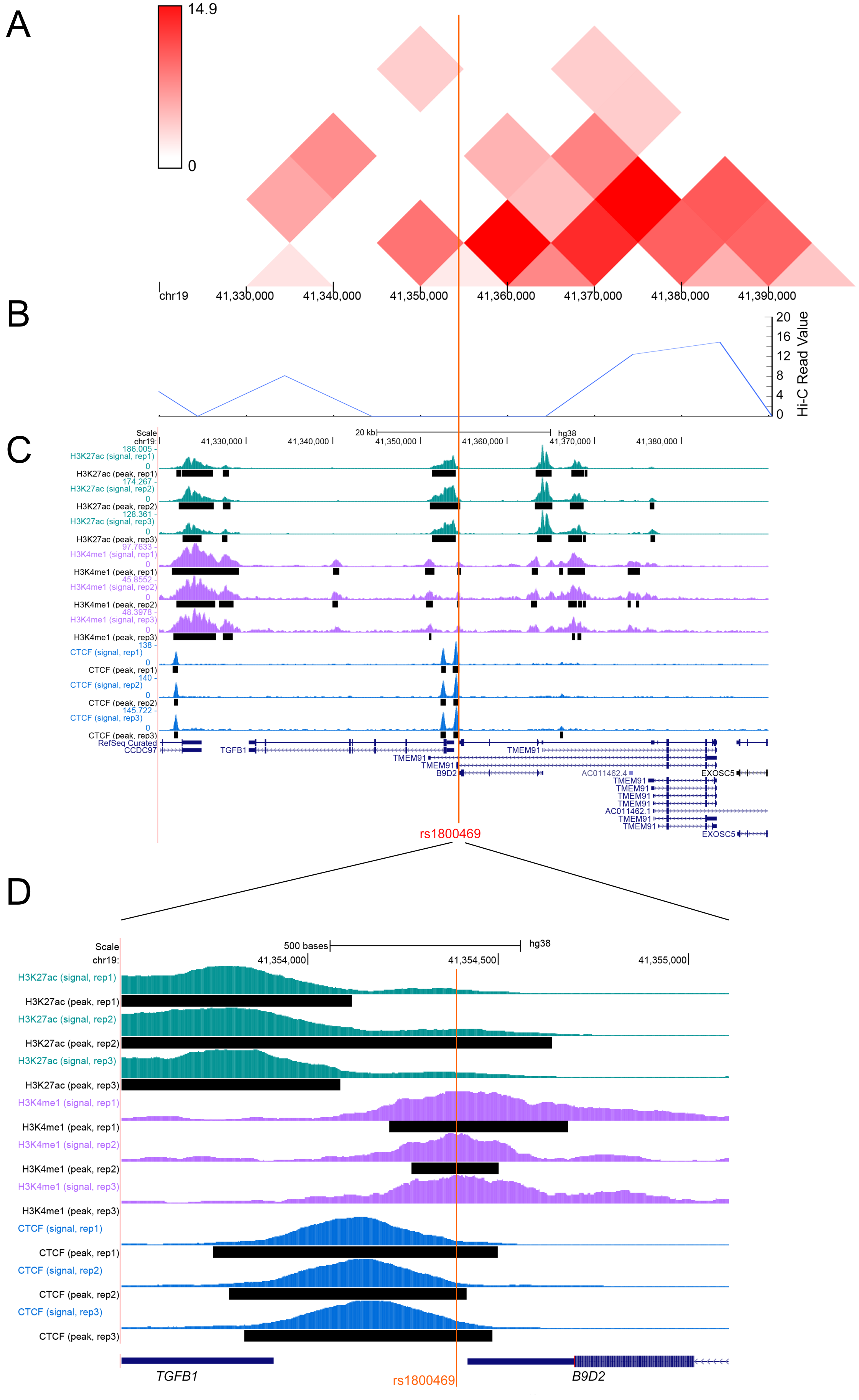
A
14.9
0
chr19
41,330,000
41,340,000
41,350,000
41,360,000
41,370,000
41,380,000
41,390,000
B
20
16
12
8
4
0
Hi-C Read Value
C
Scale
chr19:
20 kb
hg38
41,330,000
41,340,000
41,350,000
41,360,000
41,370,000
41,380,000
H3K27ac (signal, rep1)
H3K27ac (peak, rep1)
H3K27ac (signal, rep2)
H3K27ac (peak, rep2)
H3K27ac (signal, rep3)
H3K27ac (peak, rep3)
H3K4me1 (signal, rep1)
H3K4me1 (peak, rep1)
H3K4me1 (signal, rep2)
H3K4me1 (peak, rep2)
H3K4me1 (signal, rep3)
H3K4me1 (peak, rep3)
CTCF (signal, rep1)
CTCF (peak, rep1)
CTCF (signal, rep2)
CTCF (peak, rep2)
CTCF (signal, rep3)
CTCF (peak, rep3)
RefSeq Curated
CCDC97
TGFB1
TMEM91
B9D2
AC011462.4
EXOSC5
AC011462.1
rs1800469
D
Scale
chr19:
500 bases
hg38
41,354,000
41,354,500
41,355,000
H3K27ac (signal, rep1)
H3K27ac (peak, rep1)
H3K27ac (signal, rep2)
H3K27ac (peak, rep2)
H3K27ac (signal, rep3)
H3K27ac (peak, rep3)
H3K4me1 (signal, rep1)
H3K4me1 (peak, rep1)
H3K4me1 (signal, rep2)
H3K4me1 (peak, rep2)
H3K4me1 (signal, rep3)
H3K4me1 (peak, rep3)
CTCF (signal, rep1)
CTCF (peak, rep1)
CTCF (signal, rep2)
CTCF (peak, rep2)
CTCF (signal, rep3)
CTCF (peak, rep3)
TGFB1
rs1800469
B9D2

Figure S1

Figure S1. Magnified view of a non-coding intergenic region harboring SNP rs1800469 (asthma/CF/COPD modifier SNP) in A549 cells.

(A) Shown is a Hi-C result indicating interactions of genomic regions in a chromatin (chromosome) context in an A549 human lung epithelial cell line. The genomic region harboring SNP rs1800469 physically binds to the gene loci of *TGFB1*, *B9D2* and *TMEM91*. Darker red indicates higher physical interactions.

(B) Shown is a Virtual 4C image. Higher Hi-C read values indicate higher physical interactions with the region harboring SNP rs1800469.

(C) ChIP-seq image showing independent triplicates as described in Figure 1B.

(D) Magnified view of (C) demonstrating local enhancer and insulator regions.

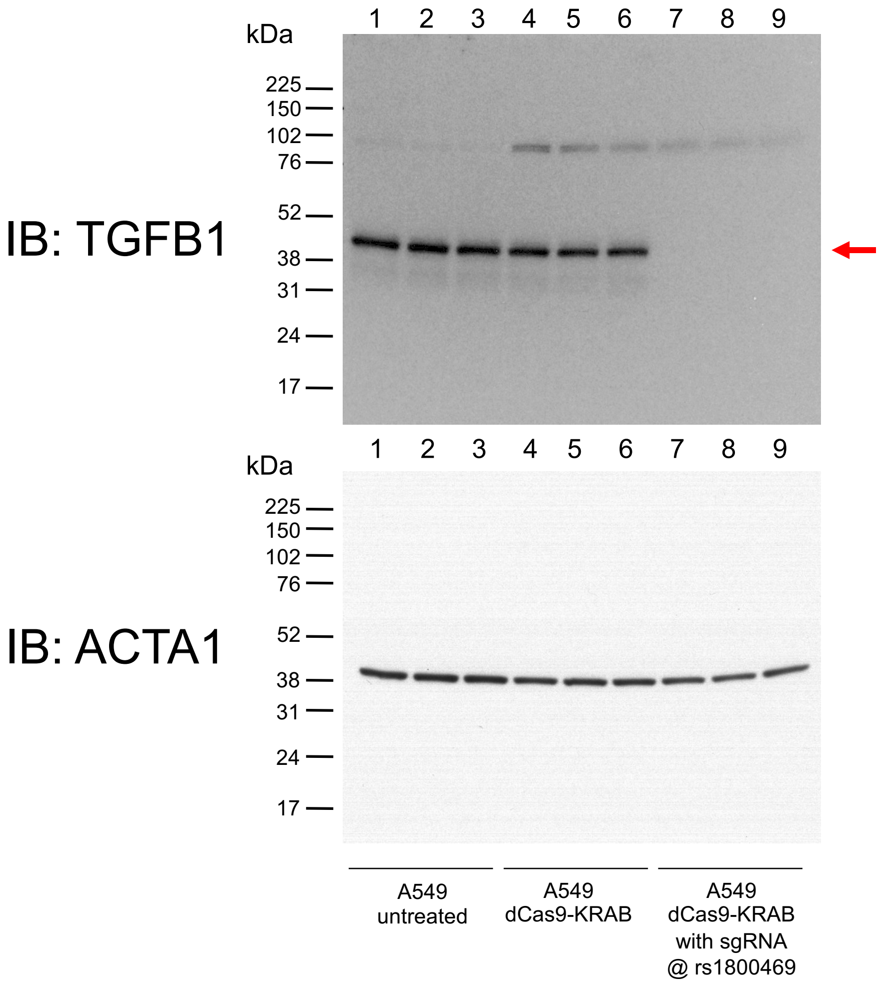

Figure S2. CRISPRi targeting a non-coding intergenic region that harbors SNP rs1800469 represses the expression of TGFB1 protein in A549 cells.

Immunoblot (IB) was performed as described using cell extracts from cell types as indicated at the bottom. Cell extracts were harvested from three independent wells for each cell type (untreated, dCas9-KRAB or dCas9-KRAB with sgRNA @ rs1800469). Shown are representative images from two independent experiments. Red arrow indicates endogenous TGFB1. The higher molecular weight band (~102 kDa) is non-specific protein. ACTA1 was used as a loading control.

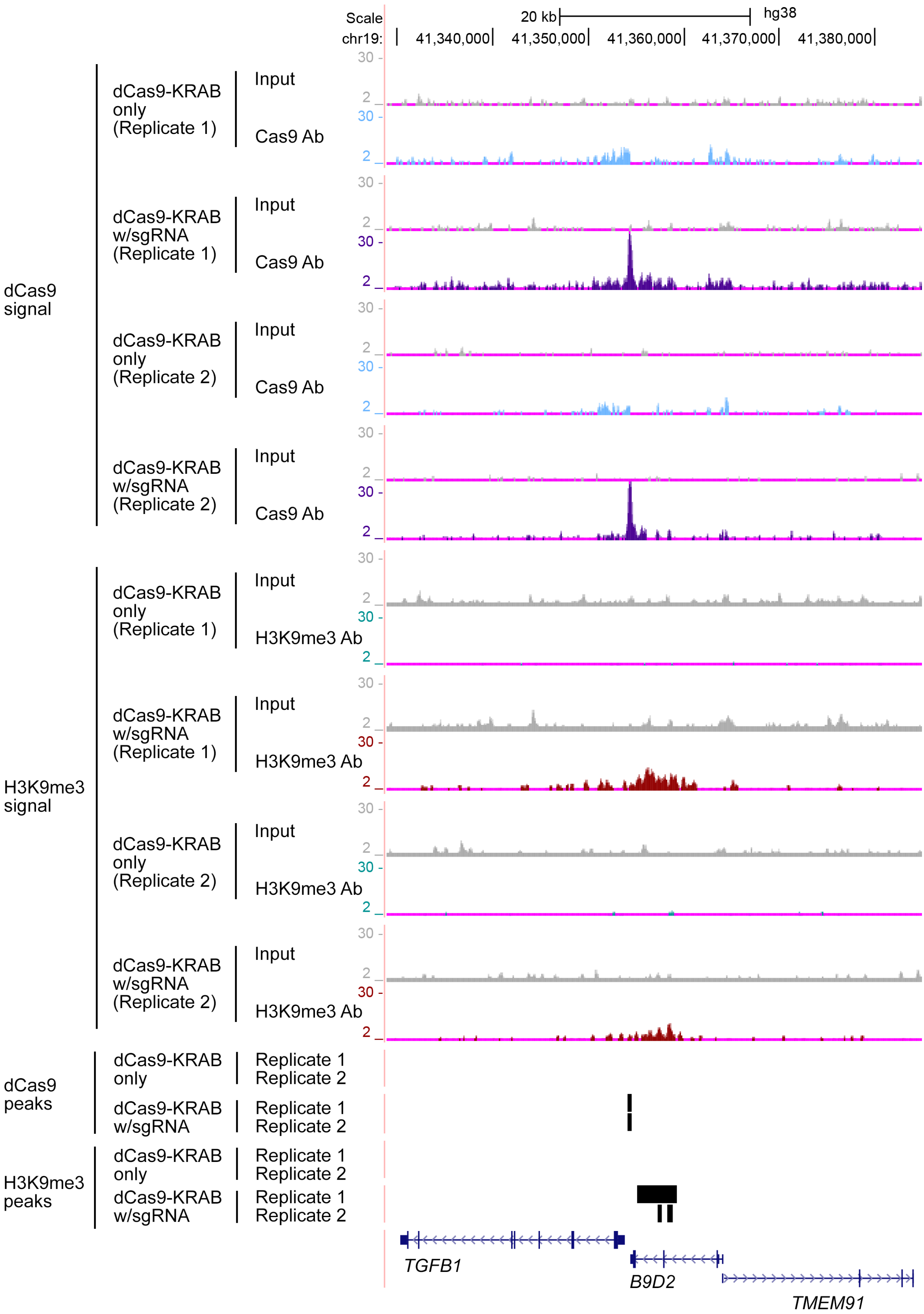

Scale
20 kb
hg38
chr19:
41,340,000
41,350,000
41,360,000
41,370,000
41,380,000
dCas9 signal
dCas9-KRAB only (Replicate 1)
Input
Cas9 Ab
dCas9-KRAB w/sgRNA (Replicate 1)
dCas9-KRAB only (Replicate 2)
dCas9-KRAB w/sgRNA (Replicate 2)
H3K9me3 signal
H3K9me3 Ab
30
2
dCas9 peaks
H3K9me3 peaks
dCas9-KRAB only
dCas9-KRAB w/sgRNA
Replicate 1
Replicate 2
TGFB1
B9D2
TMEM91

Figure S3

Figure S3. ChIP-seq indicates that the CRISPRi (dCas9-KRAB) specifically binds to the region harboring SNP rs1800469 only in the presence of the specific sgRNA and labels a repressive histone mark (H3K9me3) in H441 cells.

Shown are ChIP-seq images obtained from two independent replicates, which were in part used for Figure 3B. ChIP-seq was performed using Cas9 and H3K9me3 antibodies as described in MATERIALS AND METHODS. Bam data (top panels) indicates ChIP-seq peaks and bed data (bottom panel) indicates location of statistically significant peaks.

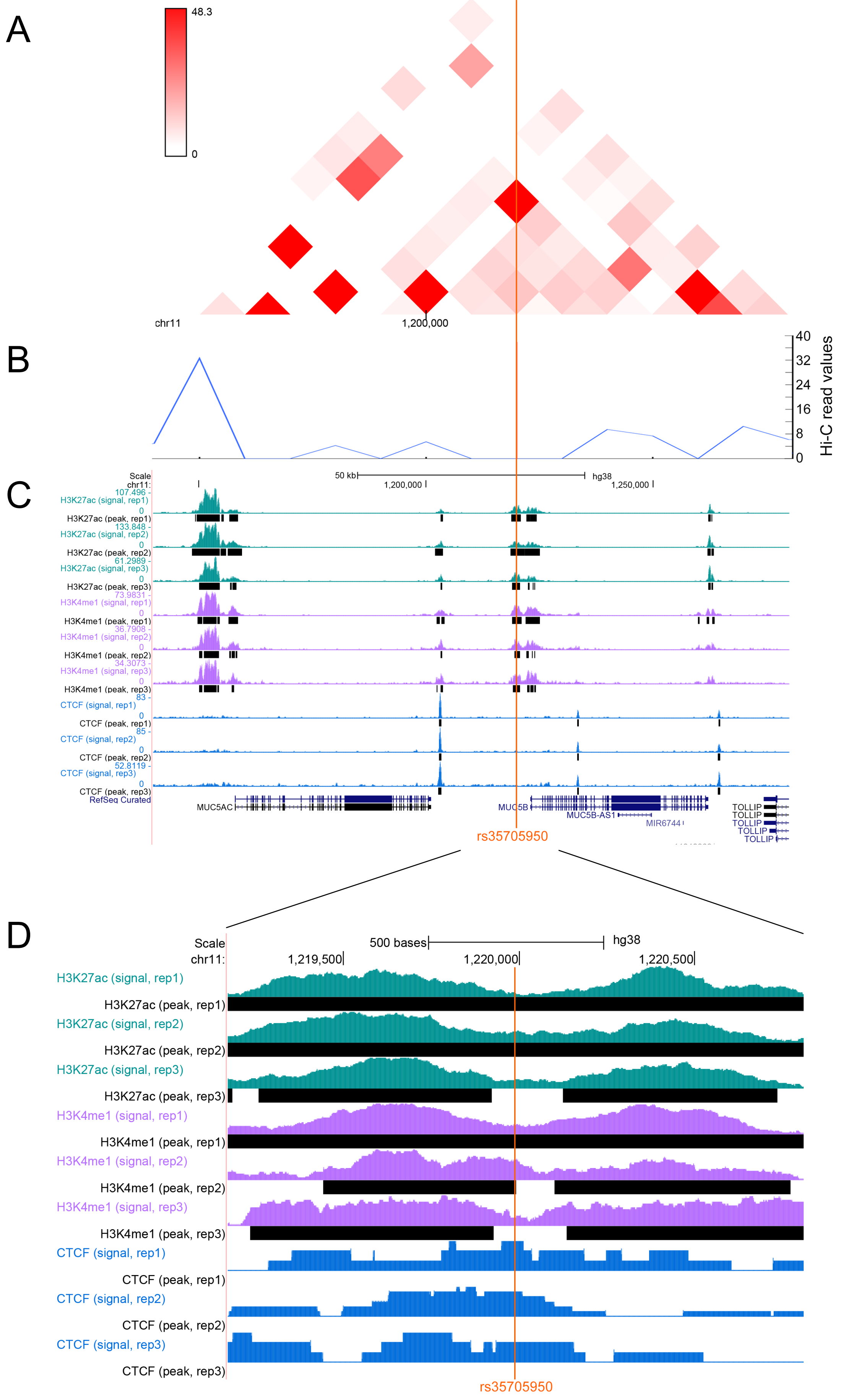
A
48.3
0
chr11
1,200,000
B
40
32
24
16
8
0
Hi-C read values
C
Scale
chr11:
50 kb
hg38
1,200,000
1,250,000
H3K27ac (signal, rep1)
H3K27ac (peak, rep1)
H3K27ac (signal, rep2)
H3K27ac (peak, rep2)
H3K27ac (signal, rep3)
H3K27ac (peak, rep3)
H3K4me1 (signal, rep1)
H3K4me1 (peak, rep1)
H3K4me1 (signal, rep2)
H3K4me1 (peak, rep2)
H3K4me1 (signal, rep3)
H3K4me1 (peak, rep3)
CTCF (signal, rep1)
CTCF (peak, rep1)
CTCF (signal, rep2)
CTCF (peak, rep2)
CTCF (signal, rep3)
CTCF (peak, rep3)
RefSeq Curated
MUC5AC
MUC5B
MUC5B-AS1
MIR6744
TOLLIP
rs35705950
D
Scale
chr11:
500 bases
hg38
1,219,500
1,220,000
1,220,500
H3K27ac (signal, rep1)
H3K27ac (peak, rep1)
H3K27ac (signal, rep2)
H3K27ac (peak, rep2)
H3K27ac (signal, rep3)
H3K27ac (peak, rep3)
H3K4me1 (signal, rep1)
H3K4me1 (peak, rep1)
H3K4me1 (signal, rep2)
H3K4me1 (peak, rep2)
H3K4me1 (signal, rep3)
H3K4me1 (peak, rep3)
CTCF (signal, rep1)
CTCF (peak, rep1)
CTCF (signal, rep2)
CTCF (peak, rep2)
CTCF (signal, rep3)
CTCF (peak, rep3)
rs35705950

Figure S4

Figure S4. Magnified view of a non-coding intergenic region harboring SNP rs35705950 (IPF modifier SNP) in A549 cells.

(A) Shown is a Hi-C result indicated as described in Supplementary Figure S1A. The genomic region harboring SNP rs35705950 physically binds to the gene loci of *MUC5AC* and *MUC5B*. Darker red indicates higher physical interactions. .

(B) Shown is a Virtual 4C image. Higher Hi-C read values indicate higher physical interactions with the region harboring SNP rs35705950 .

(C) ChIP-seq image showing independent triplicates as described in Figure 4B.

(D) Magnified view of (C) demonstrating local enhancer and insulator regions.

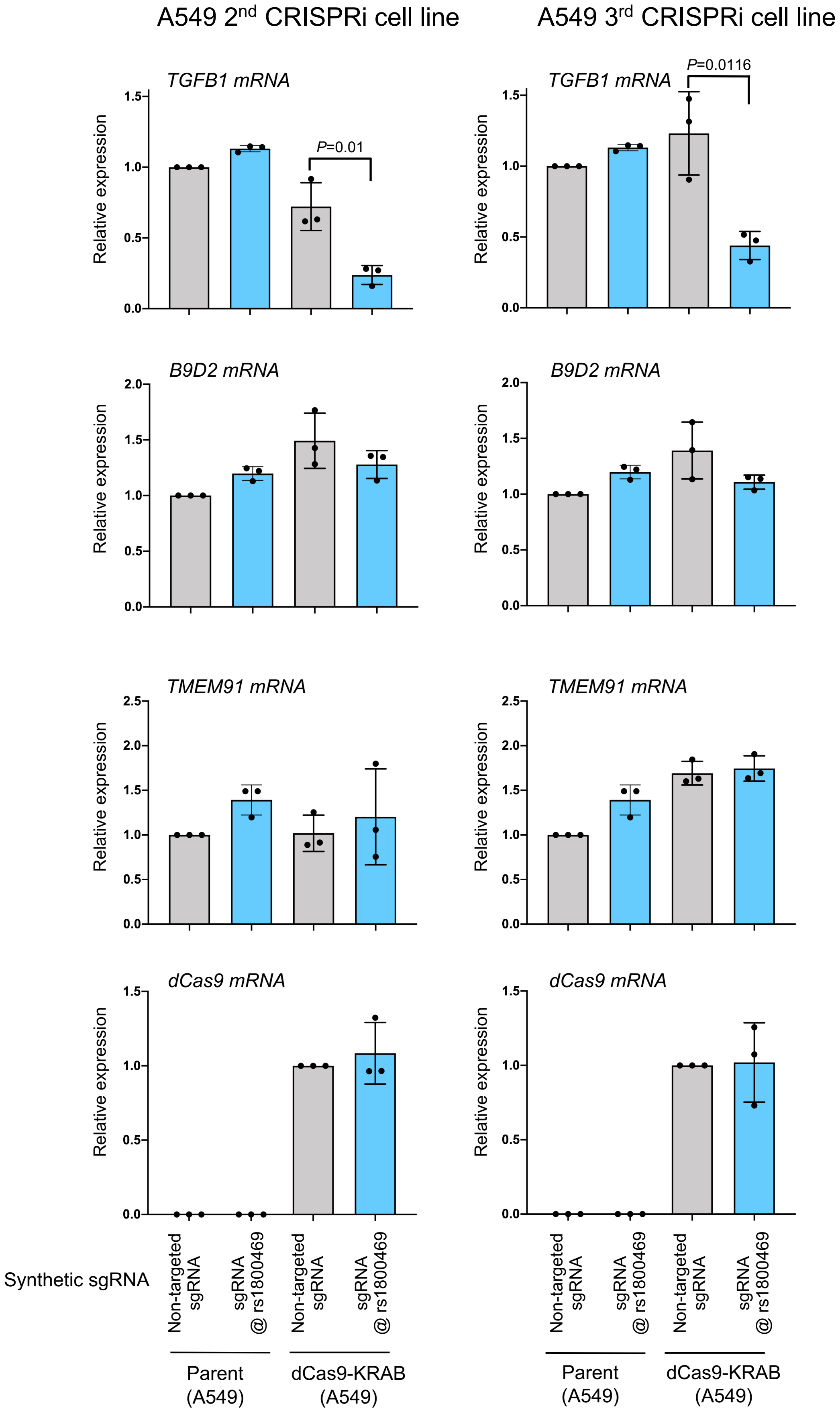
A549 2nd CRISPRi cell line
A549 3rd CRISPRi cell line
TGFB1 mRNA
P=0.01
P=0.0116
B9D2 mRNA
TMEM91 mRNA
dCas9 mRNA
Relative expression
Synthetic sgRNA
Non-targeted sgRNA
sgRNA @ rs1800469
Parent (A549)
dCas9-KRAB (A549)

Figure S5

Figure S5. Additional A549 cells stably expressing dCas9-KRAB.
Two more A549 cell lines were established by independent infection with the CRISPRi lentivirus carrying dCas9-KRAB in addition to the one used in Figures 1 and 5. These A549 cells stably expressing dCas9-KRAB were used to repeat in part the experiments performed in Figure 5. Parent cell expression data used as controls are identical in both graph sets. The data was consistent with the cell line used in Figure 5.

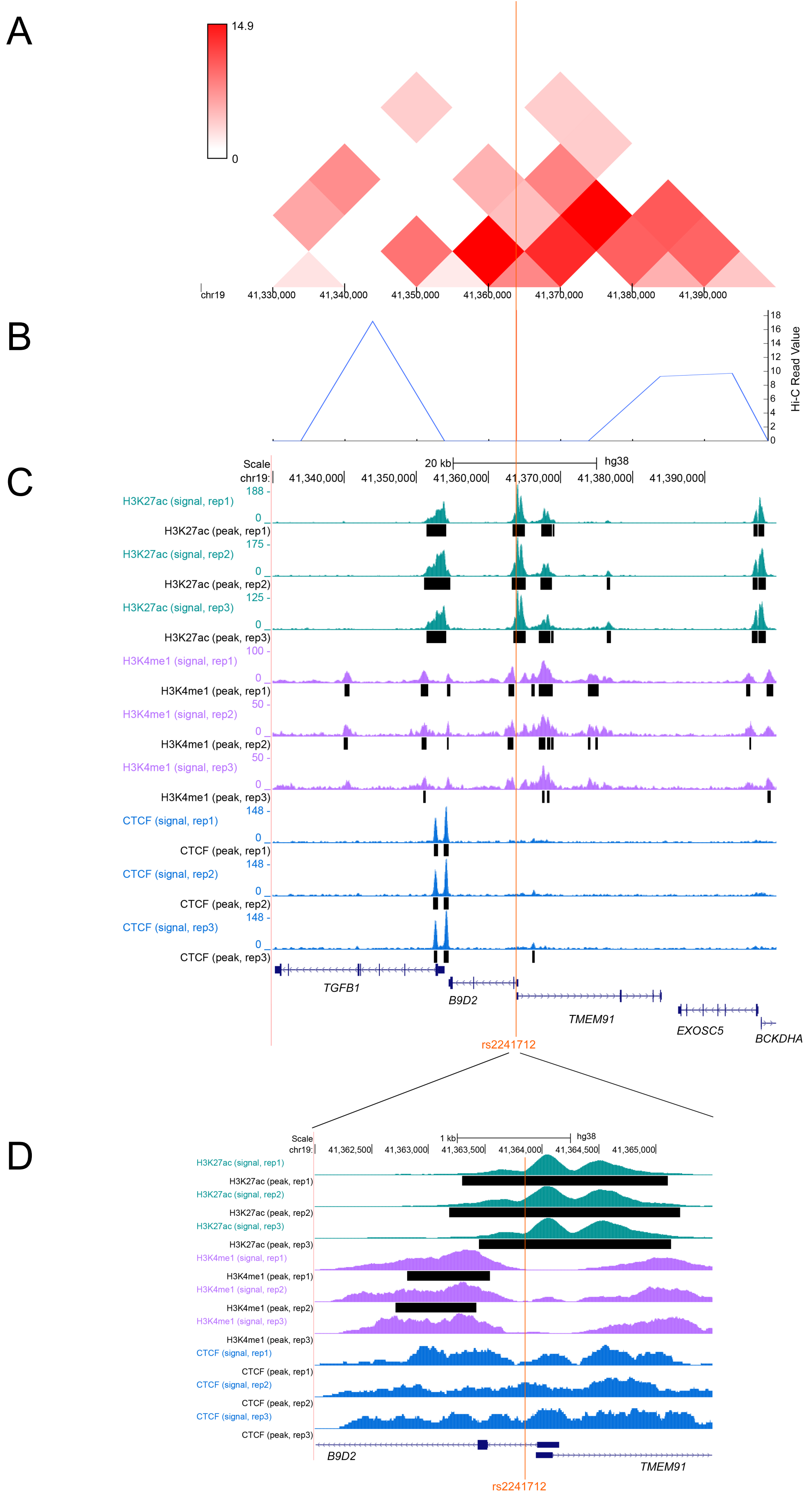

A
14.9
0
chr19
41,330,000
41,340,000
41,350,000
41,360,000
41,370,000
41,380,000
41,390,000
B
Hi-C Read Value
C
Scale
20 kb
hg38
chr19:
H3K27ac (signal, rep1)
H3K27ac (peak, rep1)
H3K27ac (signal, rep2)
H3K27ac (peak, rep2)
H3K27ac (signal, rep3)
H3K27ac (peak, rep3)
H3K4me1 (signal, rep1)
H3K4me1 (peak, rep1)
H3K4me1 (signal, rep2)
H3K4me1 (peak, rep2)
H3K4me1 (signal, rep3)
H3K4me1 (peak, rep3)
CTCF (signal, rep1)
CTCF (peak, rep1)
CTCF (signal, rep2)
CTCF (peak, rep2)
CTCF (signal, rep3)
CTCF (peak, rep3)
TGFB1
B9D2
TMEM91
EXOSC5
BCKDHA
rs2241712
D
1 kb
41,362,500
41,363,000
41,363,500
41,364,000
41,364,500
41,365,000

Figure S6

Figure S6. Magnified view of a non-coding intergenic region harboring SNP rs2241712 (asthma/COPD modifier SNP) in A549 cells.

(A) Shown is a Hi-C result indicated as described in Supplementary Figure S1A. The genomic region harboring SNP rs2241712 physically binds to the gene loci of *TGFB1*, *B9D2* and *TMEM91*. Darker red indicates higher physical interactions.

(B) Shown is a Virtual 4C image. Higher Hi-C read values indicate higher physical interactions with the region harboring SNP rs2241712.

(C) ChIP-seq image showing independent triplicates as described in Figure 6B.

(D) Magnified view of (C) demonstrating local enhancer and insulator regions.

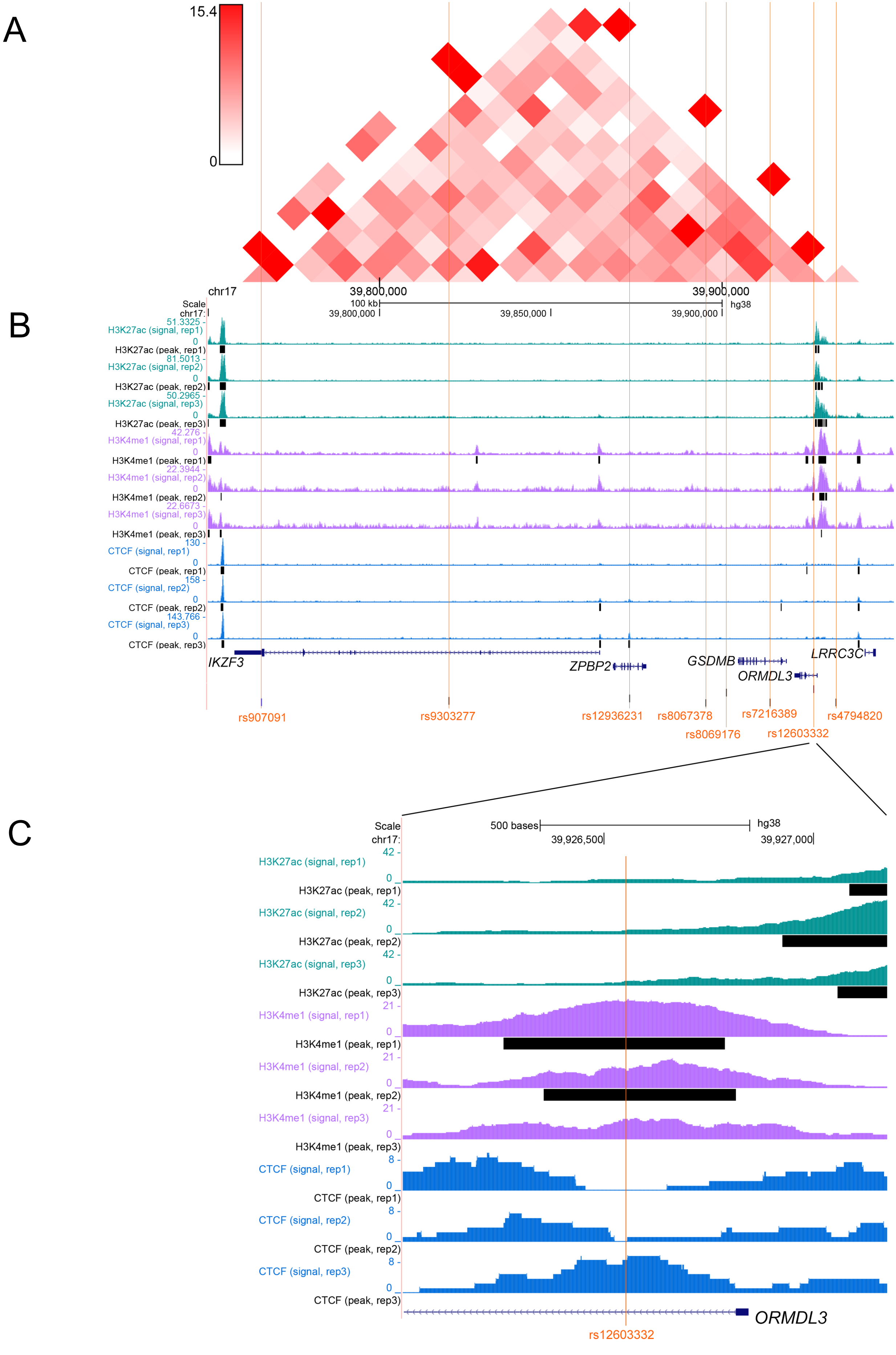
A
15.4
0
B
chr17
39,800,000
39,900,000
Scale
chr17:
100 kb
hg38
39,800,000
39,850,000
39,900,000
H3K27ac (signal, rep1)
H3K27ac (peak, rep1)
H3K27ac (signal, rep2)
H3K27ac (peak, rep2)
H3K27ac (signal, rep3)
H3K27ac (peak, rep3)
H3K4me1 (signal, rep1)
H3K4me1 (peak, rep1)
H3K4me1 (signal, rep2)
H3K4me1 (peak, rep2)
H3K4me1 (signal, rep3)
H3K4me1 (peak, rep3)
CTCF (signal, rep1)
CTCF (peak, rep1)
CTCF (signal, rep2)
CTCF (peak, rep2)
CTCF (signal, rep3)
CTCF (peak, rep3)
IKZF3
ZPBP2
GSDMB
ORMDL3
LRRC3C
rs907091
rs9303277
rs12936231
rs8067378
rs8069176
rs7216389
rs12603332
rs4794820
C
Scale
chr17:
500 bases
hg38
39,926,500
39,927,000
H3K27ac (signal, rep1)
H3K27ac (peak, rep1)
H3K27ac (signal, rep2)
H3K27ac (peak, rep2)
H3K27ac (signal, rep3)
H3K27ac (peak, rep3)
H3K4me1 (signal, rep1)
H3K4me1 (peak, rep1)
H3K4me1 (signal, rep2)
H3K4me1 (peak, rep2)
H3K4me1 (signal, rep3)
H3K4me1 (peak, rep3)
CTCF (signal, rep1)
CTCF (peak, rep1)
CTCF (signal, rep2)
CTCF (peak, rep2)
CTCF (signal, rep3)
CTCF (peak, rep3)
ORMDL3
rs12603332

Figure S7

Figure S7. Magnified view of a non-coding intergenic region harboring SNP rs12603332 (asthma modifier SNP) in A549 cells.
(A) Shown is a Hi-C result indicated as described in Supplementary Figure S1A. The genomic regions harboring asthma modifier SNPs physically bind to the gene loci of *GSDMB* and *ORMDL3*. Darker red indicates higher physical interactions.
(B) ChIP-seq image showing independent triplicates as described in Figure 7A.
(C) Magnified view of (B) demonstrating local enhancer and insulator regions.

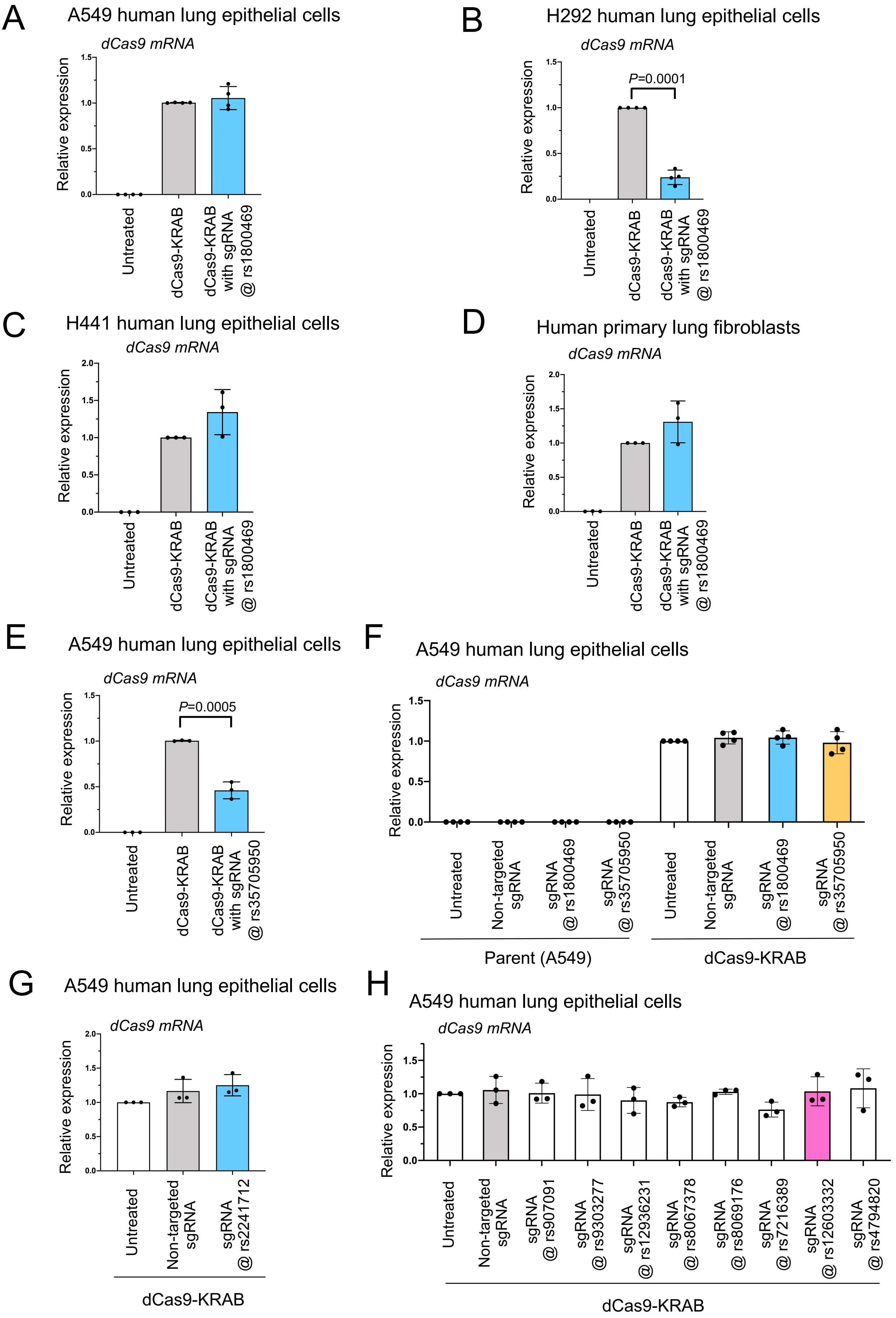
A
A549 human lung epithelial cells
dCas9 mRNA
Relative expression
1.5
1.0
0.5
0.0
Untreated
dCas9-KRAB
dCas9-KRAB with sgRNA @ rs1800469
B
H292 human lung epithelial cells
dCas9 mRNA
P=0.0001
Relative expression
Untreated
dCas9-KRAB
dCas9-KRAB with sgRNA @ rs1800469
C
H441 human lung epithelial cells
dCas9 mRNA
2.0
Relative expression
Untreated
dCas9-KRAB
dCas9-KRAB with sgRNA @ rs1800469
D
Human primary lung fibroblasts
dCas9 mRNA
Relative expression
Untreated
dCas9-KRAB
dCas9-KRAB with sgRNA @ rs1800469
E
A549 human lung epithelial cells
dCas9 mRNA
P=0.0005
Relative expression
Untreated
dCas9-KRAB
dCas9-KRAB with sgRNA @ rs35705950
F
A549 human lung epithelial cells
dCas9 mRNA
Relative expression
Untreated
Non-targeted sgRNA
sgRNA @ rs1800469
sgRNA @ rs35705950
Parent (A549)
dCas9-KRAB
G
A549 human lung epithelial cells
dCas9 mRNA
Relative expression
Untreated
Non-targeted sgRNA
sgRNA @ rs2241712
dCas9-KRAB
H
A549 human lung epithelial cells
dCas9 mRNA
Relative expression
Untreated
Non-targeted sgRNA
sgRNA @ rs907091
sgRNA @ rs9303277
sgRNA @ rs12936231
sgRNA @ rs8067378
sgRNA @ rs8069176
sgRNA @ rs7216389
sgRNA @ rs12603332
sgRNA @ rs4794820
dCas9-KRAB

Figure S8

Figure S8. Expression of *dCas9-KRAB* infected with the CRISPRi lentivirus.

The expression of *dCas9-KRAB* mRNA in A549, H292, H441 human lung epithelial cell lines and human primary lung fibroblasts infected with either control virus (*dCas9-KRAB* only) or the lentivirus carrying both *dCas9-KRAB* and sgRNA targeting the genomic regions with the SNPs was confirmed as described in MATERIALS AND METHODS. Panels represent *dCas9-KRAB* mRNA levels harvested from cells shown in Figure 1C (A), Figure 2A (B), Figure 2B (C), Figure 2C (D), Figure 4C (E), Figure 5 (F), Figure 6C (G) and Figure 7B (H).
